# Probe Electrospray Ionization (PESI) and Its Modified Versions: Dipping PESI (dPESI), Sheath-Flow PESI (sfPESI) and Adjustable sfPESI (ad-sfPESI)

**DOI:** 10.5702/massspectrometry.A0092

**Published:** 2020-12-04

**Authors:** Kenzo Hiraoka, Osamu Ariyada, Dilshadbek T. Usmanov, Lee C. Chen, Satoshi Ninomiya, Kentaro Yoshimura, Sen Takeda, Zhang Yu, Mridul K. Mandal, Hiroshi Wada, Stephanie Rankin-Turner, Hiroshi Nonami

**Affiliations:** 1 Clean Energy Research Center, University of Yamanashi, 4–3–11 Takeda, Kofu 400–8511, Japan; 2 ARIOS INC., 3–2–20 Musashino, Akishima, Tokyo 196–0021, Japan; 3 Graduate Faculty of Interdisciplinary Research, University of Yamanashi, 4–3–11 Takeda, Kofu 400–8511, Japan; 4 Department of Anatomy and Cell Biology, Faculty of Medicine, University of Yamanashi, 1110 Shimo-Kateau, Chuo, Yamanashi 409–3898, Japan; 5 Kyushu Okinawa Agricultural Research Center, National Agricultural and Food Research Organization, 496 Izumi, Chikugo, Fukuoka 833–0041, Japan; 6 Department of Chemistry, Loughborough University, Loughborough, Leicestershire LE11 3TU, United Kingdom; 7 Plant Biophysics/Biochemistry Research Laboratory, Faculty of Agriculture, Ehime University, Matsuyama 790–8566, Japan

**Keywords:** probe electrospray ionization (PESI), point analysis, surface analysis, robotic mass spectrometry

## Abstract

In 2007, probe electrospray ionization/mass spectrometry (PESI/MS) was developed. In this technique, the needle is moved down along a vertical axis and the tip of the needle touched to the sample. After capturing the sample at the needle tip, the needle is then moved up and a high voltage is applied to the needle at the highest position to generate electrospray. Due to the discontinuous sampling followed by the generation of spontaneous electrospray, sequential and exhaustive electrospray takes place depending on the surface activity of the analytes. As modified versions of PESI, dipping PESI (dPESI), sheath-flow PESI (sfPESI) and adjustable sfPESI (ad-sfPESI) have been developed. These methods are complementary to each other and they can be applicable to surface and bulk analysis of various biological samples. In this article, the characteristics of these methods and their applications to real samples will be reviewed.

## INTRODUCTION

Electrospray ionization/mass spectrometry (ESI/MS) has become an indispensable tool for analysis in many fields.^1,2)^ A variety of direct ionization methods have been developed based on ESI for the analysis of wet and dry samples. In 2001, Wachs and Henion developed a method for the direct sampling of liquid and solid samples.^3)^ This device employed a free-standing liquid junction formed *via* continuous delivery of suitable solvent which carried the extracted analyte through a pneumatically assisted electrospray capillary in front of an atmospheric pressure ionization mass spectrometer; termed as liquid extraction surface analysis (LESA) mass spectrometry.^4)^ In 2004, Cooks *et al.* developed desorption electrospray ionization (DESI), which is applicable to semi-dry and dry samples.^5)^ In DESI, a pneumatically assisted high-velocity electrospray jet is continuously directed toward the sample surface. In 2010, Roach *et al.* developed nanoelectrospray DESI (nanoDESI), an ambient method for liquid-extraction surface-sampling mass spectrometry.^6)^ This method also employed the liquid junction pioneered by Wachs and Henion.^3)^ The analytes were extracted into a solvent formed between two capillaries and the dry sample surface. One capillary supplied solvent to create and maintain the liquid bridge, and the second capillary transported the dissolved analytes from the bridge to the mass spectrometer. A high voltage applied between the mass spectrometer inlet and the primary capillary created a self-aspirating nanoelectrospray. In 2012, Otsuka *et al.* developed scanning probe electrospray ionization (SPESI), which used a solvent transport capillary as the electrospray emitter.^7,8)^ Solvent was supplied to the capillary to form a liquid bridge between the probe and the sample surface. By applying a high voltage (HV) to the capillary, an electrospray was generated from the tip of the capillary. Because the extraction of the analytes at the liquid bridge and ESI of the solution occurred around the probe tip, transportation of the sample solution through a secondary capillary for ESI was not necessary. In 2016, Ji *et al.* reported a ballpoint electrospray ionization mass spectrometry (BP-ESI-MS) technique.^9)^ This combined a small ballpoint tip with a syringe pump for the direct loading and ionization of various samples in different phases including solution, semisolid, and solid. The rigid properties of the ballpoint tip allowed sampling by simply penetrating or scraping various surfaces. In 2015, Rao, Pan and Yang developed a miniaturized sampling and ionization device, single probe mass spectrometry (Single-probeMS), which used *in-situ* surface micro-extraction to achieve high detection sensitivity and spatial resolution mass spectrometry.^10)^ The single-probe consisted of a dual-bore quartz probe, a fused silica capillary, and a nano-electrospray emitter. By positioning the single-probe tip above the sample surface, microextraction took place at the tip of the probe. The continuous flow of sampling solvent produced a consistent fresh liquid junction at the probe tip, allowing for constant extraction of analytes present on the surface. A spatial resolution of 8.5 μm was achieved in the analysis of biological tissues. For all the techniques described above, a liquid junction between the probe tip and the sample surface was achieved by using a “continuous” flow of solvent through the capillary.

In 2007, a “discontinuous” sampling and electrospray ionization method, probe electrospray ionization (PESI), was developed.^11)^ PESI is free from clogging problems and is suitable for direct analysis of various wet biological samples, including those with a high salt concentration.^12)^ The great merit of the discontinuous sampling/electrospray is that the ion suppression effect was largely moderated in PESI because of the occurrence of sequential and exhaustive electrospray.^13)^ Modified versions of PESI, dipping PESI (dPESI)^14)^ sheath-flow PESI (sfPESI)^15)^ and adjustable sfPESI (ad-sfPESI)^16)^ were subsequently developed. In dPESI, the sample surface is pricked with a fine bare acupuncture needle and the sample is captured at the needle tip. After drying the sample, the needle tip is dipped into the pure solvent for ∼50 ms and the wetted needle moved upward. At the highest position of the needle, a HV is applied to the needle to generate electrospray. In sfPESI, an acupuncture needle is inserted into a fine plastic capillary with a protrusion of 0.1–0.2 mm out of the tip. Analytes are extracted by filling the capillary with solvent and softly touching the sample surface for a short time (50 ms∼a few s). By applying a HV to the acupuncture needle, mass spectra of analytes are obtained by “self-aspirating” electrospray. In ad-sfPESI, the sample surface is pricked with an acupuncture needle inserted in the sfPESI probe that protrudes from the terminus of the tip by 5 mm. The invasion depth of the needle into the sample is ∼1 mm. After sampling, the needle is retracted into the solvent-preloaded capillary with a protrusion length of 0.1–0.2 mm from the tip. A mass spectrum of the sample captured on the needle is then obtained as in the case of PESI and dPESI.

The limitation of PESI, dPESI and ad-sfPESI is that they are difficult to apply to dry samples directly and some additional sample preparation is necessary. In contrast, sheath-flow PESI (sfPESI) is readily applicable to dry samples as well as liquid and wet samples.^15)^ In this report, the advantages and disadvantages of PESI, dPESI, sfPESI and ad-sfPESI will be described for the practical applications of the methods.

## WHAT IS ELECTROSPRAY?

**Figure 1:**
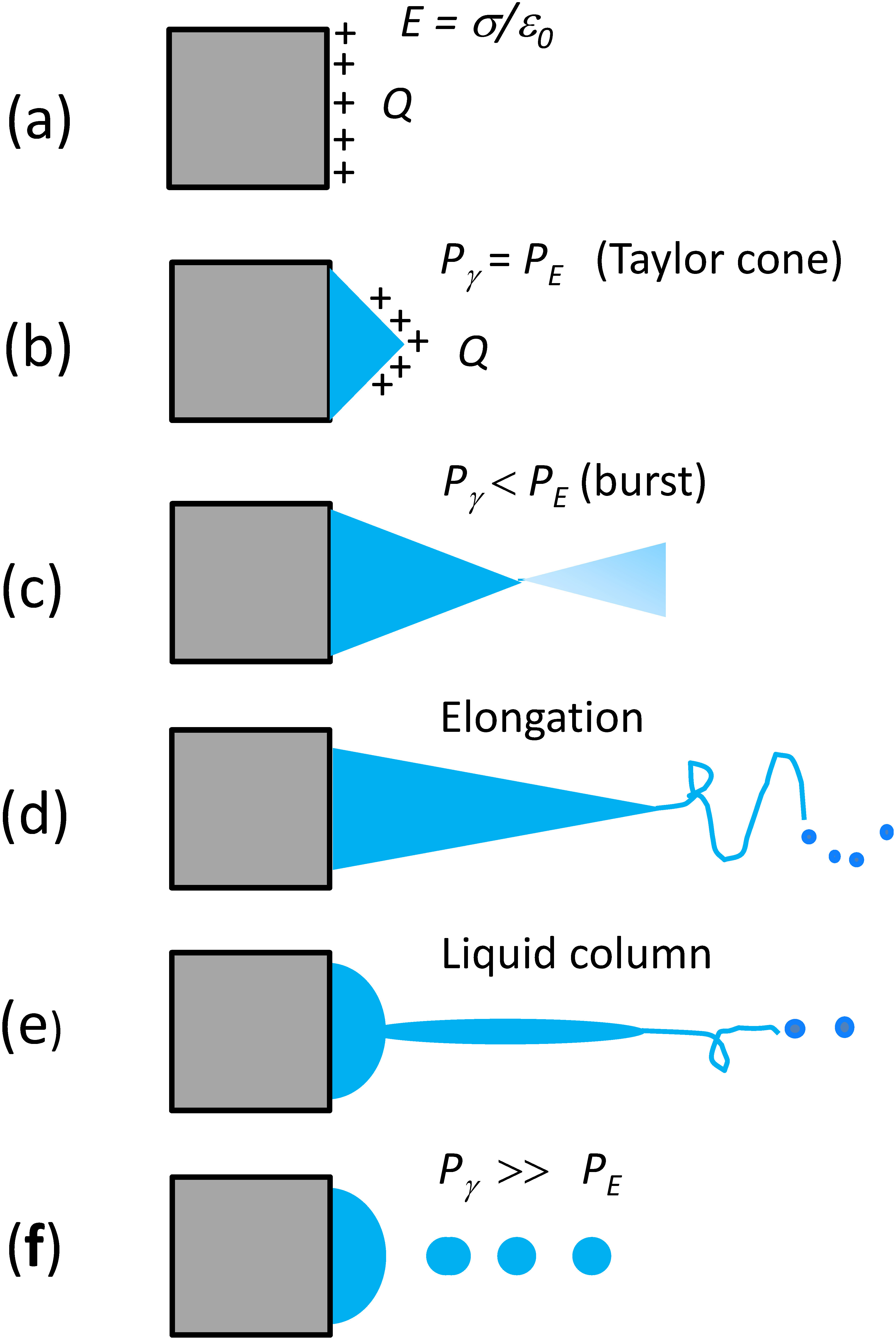
Fig. 1. Evolution of electrospray. (a) Metal capillary with the application of a HV. (b) Excess charge *Q* transferred from the metal to the surface of the liquid. (c) Burst of the Taylor cone. (d) and (e) Elongation of the Taylor cone. (f) Spherical liquid after the loss of excess charge.

The electrospray ionization (ESI) process is the action of electrolytic liquid dispersion into a fine aerosol, a phenomenon that takes place when a strong electric field is exerted on the liquid. Figure 1(a) shows the metal capillary with the application of a HV. The strong electric field *E* is generated at the tip of the metal capillary. To a first approximation, *E* is inversely proportional to the radius of the curvature. That is, the strongest *E* is generated at the capillary tip. The electric field *E* is proportional to the surface charge density σ as shown in Eq. (1).
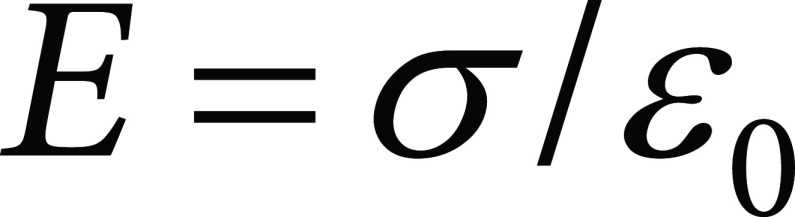
(1)

When a liquid is supplied to the capillary (Fig. 1(b)), the excess charge *Q* induced on the metal tip is transferred to the surface of the liquid due to the electrochemical reactions taking place at the interface between the metal and the liquid. With the excess charge *Q* in the droplet increasing, the shape of the liquid becomes conical because of the Coulomb’s repulsive forces acting normal to the surface in the outward direction. On the other hand, to minimize the surface area of the liquid, the liquid surface tension acts normal to the surface as the inward force. At the Rayleigh limit (Fig. 1(b)), the outward and inward forces balance each other at all positions in the liquid and the whole angle of 99° is formed.

The pressure *P_γ_* originating from the surface tension of the liquid (inward pressure normal to the surface) is proportional to the product of the surface tension γ and inversely proportional to the curvature radius *r*.
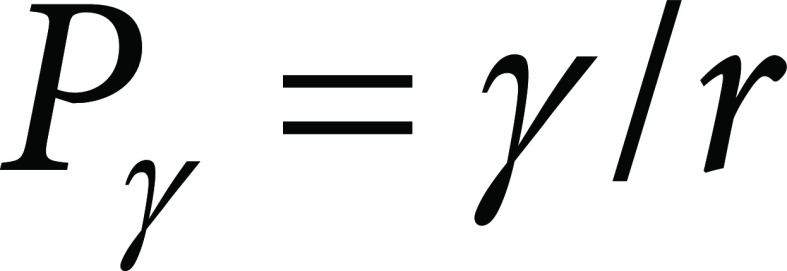
(2)

The electrostatic pressure *P_E_* induced on the surface of the liquid by the excess charges is proportional to *E*^2^.
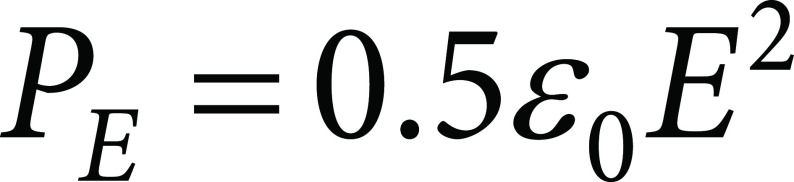
(3)

The critical voltage *V_c_* for the formation of the Taylor cone with *P_E_*=*P_γ_* is given by Eq. (4) where *d* is the distance between the needle tip and the counter electrode.
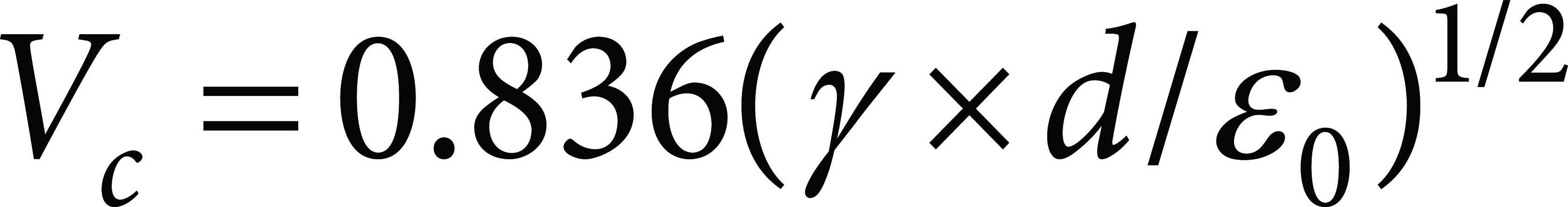
(4)

With further increase in a HV over the Rayleigh limit, *P_E_* becomes larger than *P_γ_* and the Taylor cone elongates to the counter electrode.^17)^ Figure 1(c) shows the burst of the Taylor cone right after the collapse of the Taylor cone. Due to the Coulomb explosion, numerous charged fine droplets are liberated from the tip of the Taylor cone. Accompanied with the explosion, the excess charges in the droplet at the tip decrease suddenly and the speed of the elongation of the Taylor cone slows down resulting in the formation of a long liquid column (Fig. 1(d)→(e)). The elongated liquid column is divided into much larger droplets (Fig. 1(f)) than those in Fig. 1(c). By the loss of excess charges, *P_γ_* overwhelms *P_E_* and the meniscus of the liquid becomes spherical (Fig. 1(f)). As shown in Fig. 1, electrospray generates pulsed charged liquid droplets with a wide size distribution. The pulsation of electrospray is referred to as “electric sneezing.” The time necessary for one episode of electric sneezing (*i.e.*, liberation of excess charge and the provision of charge to the sample solution by electrochemical reactions) was measured to be ∼100 s of μs.^18,19)^ That is, electrospray is generated periodically with the frequency of a few kHz with the repetitive cycle of *P_γ_*≶*P_E_*.

Because the charged droplets are generated with the conditions of *P_γ_*<*P_E_* from the tip of the Taylor cone, all the charged droplets have the excess charges *Q* that are close to the Rayleigh limit regardless of the sizes of the droplets.
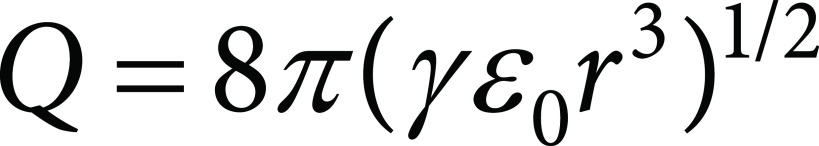
(5)

Here, *Q* is the excess charge at the Coulomb instability for the liquid droplet with the radius of *r*.

## DOWNSIZING OF ELECTROSPRAY

The electrospray current (*i*=d*Q*/d*t*) represents the rate of electrochemical reactions taking place at the interface between the metal electrode and the liquid. The electrospray current is dependent on various factors such as the size of the capillary electrode, applied voltage, distance toward the counter electrode, components of solution, and the solvent, but generally it is more or less of the order of a few hundreds of nA. Therefore, the excess charge *Q* supplied to the unit volume of the solution increases as the flow rate of the solution is decreased. It is evident that nanoelectrospray (nESI) that deals with low flow rate gives a high analytical performance in ESI/MS.

The excess charge *Q* in Eq. (5) in the droplet exists on the surface of the charged droplet (no electric field on the inside of the droplet). If one divides *Q* by the surface area of the spherical droplet with radius *r*, the surface charge density, σ=*Q*/4π*r*^2^=2(ε_0_γ)^1/2^/*r*^1/2^, is obtained. That is, the surface charge density σ is inversely proportion to *r*^1/2^. In other words, the surface charge density σ will increase with the decrease of the size of the charged droplet resulting in the higher ion detection sensitivity in ESI/MS.

**Figure 2:**
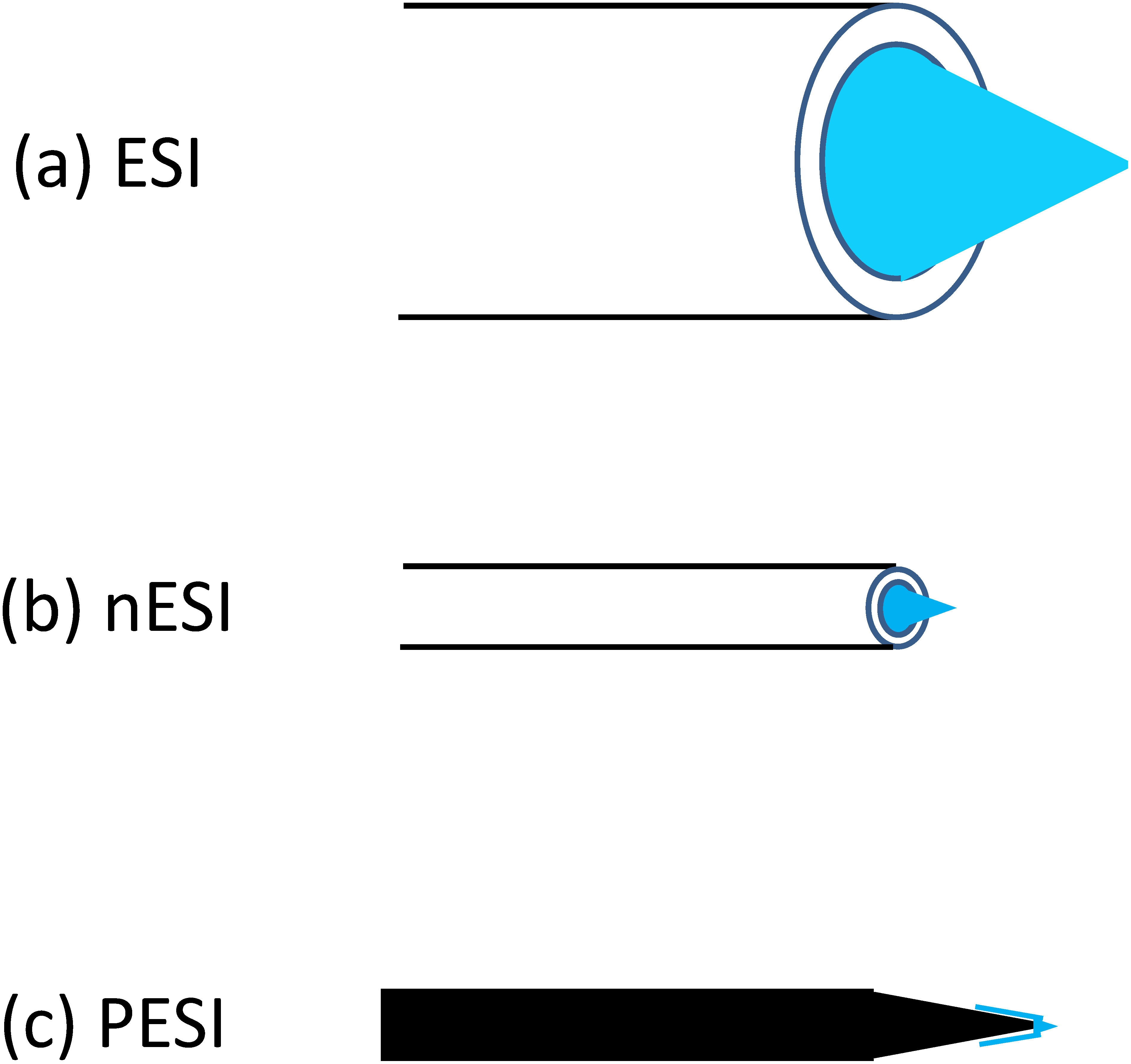
Fig. 2. (a) Electrospray using a capillary with an inner diameter of ∼0.1 mm. (b) Electrospray using a capillary with an inner diameter of a few μm. (c) Probe electrospray using a sharp metal needle.

In 1984, Yamashita and Fenn detected multiply-protonated peptides and proteins using a capillary with an inner diameter of 0.1 mm^20)^ (Fig. 2(a)). In 1994, Wilm and Mann reported that the ion detection sensitivity increased dramatically by reducing the inner diameter of the capillary^21,22)^ (Fig. 2(b)). One disadvantage of nESI is the clogging of the capillary, thus the inner diameter of a few μm may be the lower limit for the practical use of nESI. To solve the clogging problem for nESI, probe electrospray ionization (PESI) was developed in 2007 (Fig. 2(c)). As described below, PESI uses a sharp metal needle instead of a capillary.

## PROBE ELECTROSPRAY IONIZATION (PESI)

### PESI system

**Figure 3:**
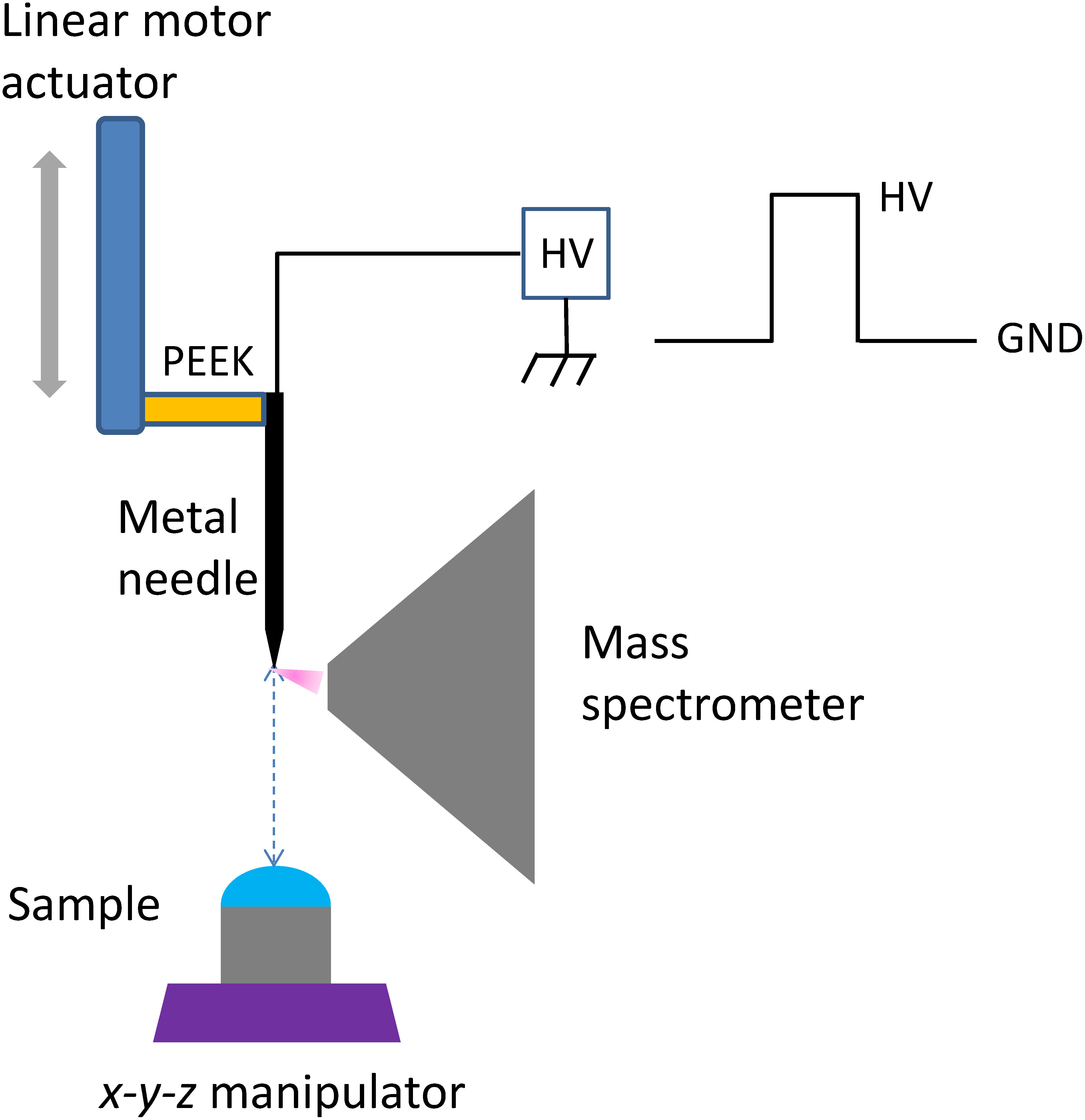
Fig. 3. Schematic diagram for the probe electrospray ionization. The perpendicular up-and-down motion of the needle was driven by a linear motor actuator. The metal needle is supported by the insulating PEEK arm.

The conceptual idea of the PESI system is shown in Fig. 3. As one example, a stainless steel acupuncture needle with a submicrometer tip diameter of ∼700 nm is used as an electrospray emitter. The metal needle (*i.e.*, the probe) is moved up and down along a vertical axis using a linear motor-actuated system. The bottom position of the needle tip is adjusted to just touch the surface of the sample that is mounted on the *x-y-z* manipulating stage. The invasion depth of the needle into the sample should be 0.5 mm or less, which is enough to obtain strong enough ion signals. When the needle is in motion and comes into contact with the sample, both the needle and the sample are kept at ground or floating potential for the safety of the operators. For liquid samples as shown in Fig. 3, the liquid meniscus should have a convex shape rather than concave one. This is because a liquid with a convex shape has a positive pressure compared to the atmospheric pressure and thus the liquid can be more effectively captured by the needle tip. The contact time of the needle tip with the sample is ∼50 ms. The surface of the needle should be clean and hydrophilic to make it wettable with the liquid sample. When the needle is contaminated with a hydrophobic insulating film, electrospray becomes unstable. The spoiled needle should be replaced or cleaned by ultra-sonication using organic solvents such as alcohols or acetonitrile. To generate electrospray, a high voltage of 1–3 kV is applied to the needle when it is moved to the highest position in front of the inlet of the mass spectrometer. The PESI mass spectrum is obtained by single-shot sampling. Because only a small sample amount is required, PESI is suitable for the analysis of very small or precious samples such as forensic or archaeological samples.

For conventional capillary-based electrospray, careful adjustment of the applied voltage to the capillary is necessary to avoid the generation of multiple-cone electrospray jets emitted from the rim of the capillary tip. In contrast in PESI, stable *single-cone* electrospray is generated from the tip of the needle. This is because the strongest electric field is generated at the apex of the sharp needle.

As for the PESI needles, any kind of metals such as micro-needles for scanning tunneling microscopy, sewing needles, sharp W/Ni/Ti/Au/Pt wires can also be used.^12)^ Titanium needles are the most useful to observe the sequential and exhaustive electrospray, probably because of its rough surface. Typically, we use an acupuncture needle (J type No. 2, SEIRIN Co., Ltd., Shizuoka, Japan) with a body and tip diameter of 0.12 mm and ∼700 nm, respectively.

### Sample amount

The liquid amount captured on the acupuncture needle tip has been previously measured.^23)^ When dipped to a depth of approximately 8 μm, the volume of the liquid was determined to be 0.4 to 5.7 pL, depending on the sample type. The general trend was that the volume of captured liquid was dependent on the viscosity and surface tension of the samples. For non-viscous samples such as aqueous solutions (*e.g.*, urine), a liquid film with a thickness in the of order of μm was captured on the surface of the stainless steel needle. In the common operation of PESI with an invasion depth of 0.5 mm into the samples, strong ion signals could be obtained for samples such as urine, serum, mouse liver and brain.^23,24)^ By using an acupuncture needle, the electrospray lasted less than 1 s with the invasion depth of ∼0.5 mm.

### Suppression of the occurrence of corona discharge

The electric field *E* at the needle tip with the curvature radius *r* is given by Eq. (6) where *V* : applied voltage, *d* : distance between the tip and the counter electrode.^25)^
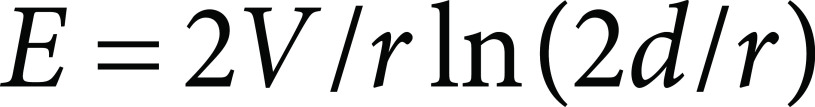
(6)

For the typical experimental conditions, *e.g.*, *d*=3 mm, *r* for the acupuncture needle=700 nm, and *V*=2×10^3^ V, *E* is roughly estimated to be 10^8^ ∼ 10^9^ V/m. This *E* is orders of magnitude larger than the threshold value (∼10^6^ V/m) for the gas breakdown (*i.e.*, occurrence of corona discharge). However, corona discharge is seldom observed under the normal conditions for positive-mode PESI. This is because the needle tip is covered by the liquid. The breakdown voltage in the liquid phase is orders of magnitude higher than that in the gas phase. In negative-mode PESI, however, careful adjustment of *V* and *d* is necessary to suppress the occurrence of corona discharge because of the electron emission from the tip due to the tunneling effect.^25)^

### Occurrence of sequential and exhaustive electrospray

**Figure 4:**
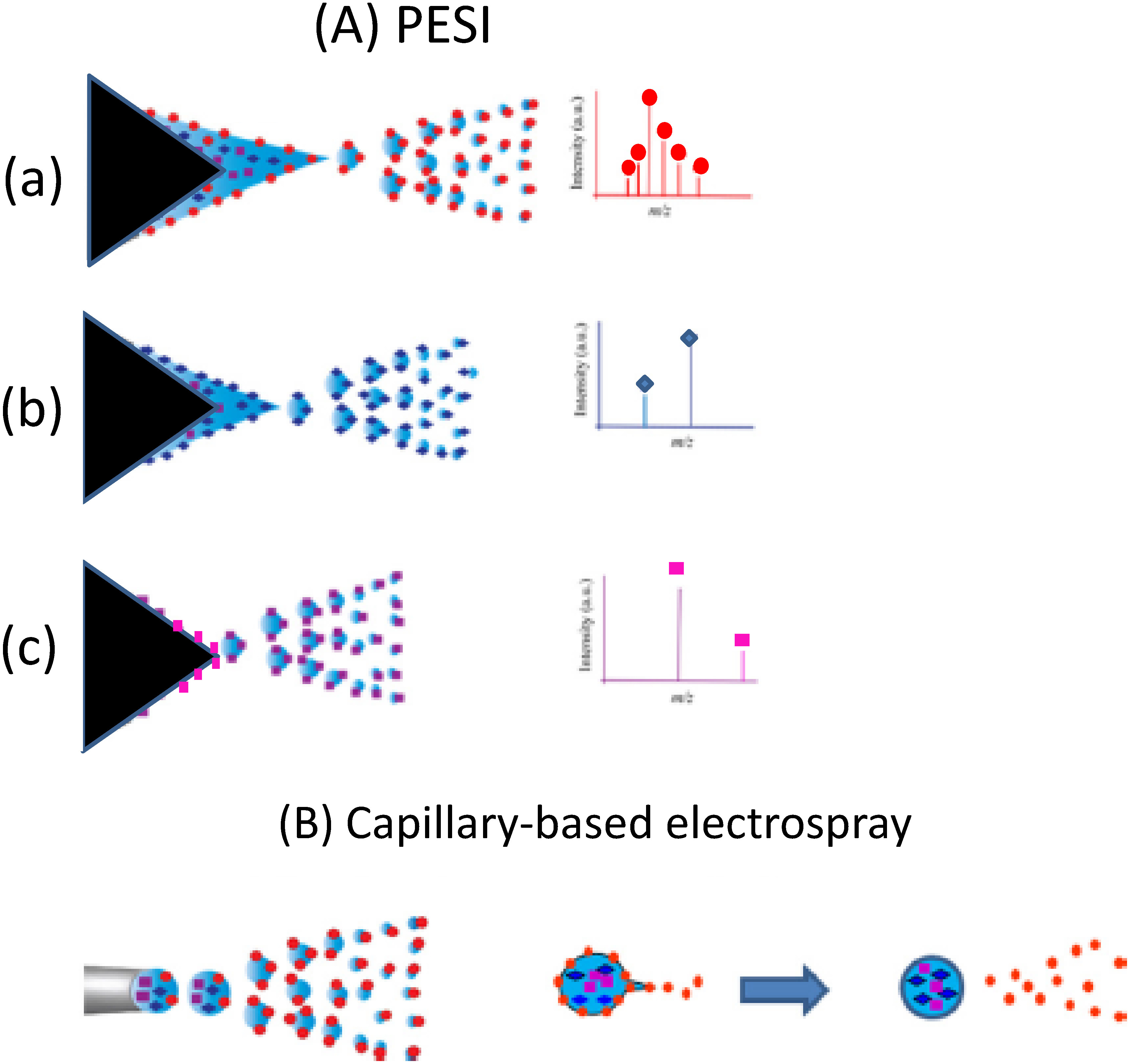
Fig. 4. (A) Probe electrospray ionization for the sample containing three analytes with different surface activities. (B) Capillary-based electrospray for the sample containing three analytes with different surface activities.

In conventional capillary-based electrospray that uses a liquid pump, liquid sample flows continuously, *i.e.*, *forced* electrospray takes place. In contrast, PESI relies on discontinuous sampling followed by spontaneous electrospray. Thus, the flow rate of the liquid is solely determined by the consumption of liquid by spontaneous electrospray itself. A mass spectrum for the sample can be obtained with only one single-shot sampling. The sample amounts necessary for PESI range from subpicoliters to nanoliters depending on the needle thickness and the invasion depth of the needle into the sample. The s*ingle-shot* electrospray gives unique features for PESI. Mandal *et al.* found that analytes were electrosprayed *sequentially* in the order of their surface active values.^13)^ Figure 4(A) shows the conceptual idea for the occurrence of sequential and exhaustive ionization in PESI for a sample solution composed of three analytes with different surface active values. Because the excess charges are continuously supplied to the liquid by the electrochemical reactions, three analytes are electrosprayed sequentially in the order of their surface active values until all samples are exhausted. In the process in Fig. 4(a)→(b)→(c), while the surface tension of the liquid (*i.e.*, *P_γ_*) increases because a less-surface active analyte has larger surface tension, the electric field at the needle tip (*i.e.*, *P_E_*) also increases with the liquid becoming thinner. Figure 4(B) shows the capillary-based electrospray. After detaching from the capillary, no excess charges are supplied to the droplet. Thus less surface-active analytes are apt to be left in the primary droplet and the suppression effect is unavoidable.^26,27)^

For example, for a mixed sample of 10^−3^ M Triton X100 (detergent) and 10^−5^ M cytochrome *c*, the detergent and protein were detected separately and respectively at the first and last stages of electrospray in PESI.^27)^ For human breast cancer tissues, at first multiply-protonated proteins such as α and β chains of hemoglobin were detected as the dominant ions. But only sodiated lipids were detected just before the liquid droplet on the needle was depleted.

The high tolerance of PESI to solutions of high salt concentration was demonstrated by its application to high-concentration aqueous alkaline copper(II)-lactate solutions.^28)^ For the mixed aqueous solution of 0.4 M copper(II)−3.0 M lactate and 3.7 M NaOH, cluster ions of [Cu+(lactate−H)_2_]^2−^ were clearly detected as Na_3_[Cu(lactate−H)_2_]^+^⋅⋅⋅⋅[Na+lactate]*_x_* (*x*=1,2,3,…). That is, in the Cu^2+^ complex, two doubly deprotonated lactic acids (*i.e.*, two [lactate−H]^2−^) interact with the core ion Cu^2+^ as ligands.

### Depth profile

By changing the invasion depth of the needle into the sample, qualitative information on the depth profile can be obtained. For example, PESI mass spectra for mouse brain were measured by changing the invasion depth of the needle step-by-step with 2.5 μm intervals.^29)^ It was found that the mass spectra were highly dependent on the depth of the sample stuck by the needle. For example, phosphatidylcholine [PC34: 1+H]^+^ was detected as a base peak at the surface of the sample. With the increase of the probing depth into the sample, the intensity of the potassiated phosphatidylcholine [PC34: 1+K]^+^ increased and became stronger than that of [PC34: 1+H]^+^. Strong ion signals were obtained with the size of the hole being a few μm and with an invasion depth of ≈10 μm. To a certain extent, the results contained information that reflects vertical distribution of analytes beneath the sample surface. However, due to the cone shape of the needle tip, the hole became bigger as the needle penetrated deeper. Since the obtained mass spectrum at a particular sampling depth could also be attributed to the ions from the previous depth, the interpretation of results may not be straightforward.^30)^ For the application of PESI to the precise depth analysis, some additional technique should be incorporated (*e.g.*, cryogenic dissection).

### Application to reaction monitoring

By repeating the single-shot PESI operation, monitoring of chemical reactions is one of the most interesting applications of PESI. Most methods for monitoring enzyme-catalyzed reactions are based on changes in spectroscopic properties during the conversion of substrates to products. Yu *et al.* employed PESI to monitor some typical protease-catalyzed reactions: pepsinolysis and trypsinolysis of cytochrome *c* in real time.^31)^ Peptic and tryptic digestion of cytochrome *c* showed different and characteristic catalytic pathways. Pepsin preferentially cleaves peptide bonds on the surface of a substrate and peptide bonds on the inner parts will become accessible resulting in the whole reactions multi-staged. In contrast, trypsin is often notated as an opposite example to pepsin due to its high but narrow specificity. The high specificity makes trypsin the first choice protease for most MS-based proteomics.

Yu *et al.* also applied PESI to monitor some biological and chemical reactions in real-time, such as acid-induced protein denaturation, hydrogen/deuterium exchange of peptides, and Schiff base formation.^32)^ PESI/MS can be considered as a potential tool for real-time reaction monitoring due to its simplicity in instrumental setup, direct sampling with minimum sample preparation and low sample consumption.

### Biomedical applications

PESI is one of the most promising techniques in biomedical analysis because it is possible to analyze biological samples very quickly without any special pretreatment. By using a needle with a very sharp tip, PESI has advantages such as low invasiveness to the samples, making it possible to analyze the biological profiles of organs or tissues in living animals *in situ*. Yoshimura *et al.* performed real-time analysis of living mice that delineated the differences in lipid composition of hepatocytes between normal and steatotic mice.^33)^ In steatotic mice, the number of peaks and the ion abundance for triacylglycerols were much higher than those of controlled mice. All mice used in that study tolerated the procedure well and survived for more than a month until being sacrificed for further analysis. To investigate the potential for medical diagnosis, Yoshimura *et al.* examined human tumor tissues and they obtained discriminative results judged as useful for diagnostics.^34)^ Now PESI/MS is being developed into a cancer diagnostics system based on mass spectrometry and machine learning (DPiMS-2020, Shimadzu Co.).^35,36)^

### Imaging mass spectrometry

Because the sampling area is determined by the needle probe dimensions, a lateral resolution of <100 μm or much smaller can be achieved if PESI is applied to imaging mass spectrometry. However, to construct reliable mass spectrometric images, several problems have to be solved. First, for relatively dehydrated surfaces, the amount of biological fluid adhering to the needle is so little that it could dry out quickly before the electrospray is initiated. Second, because sampling is performed by pure physical means, the carry-over of analytes from one sampling spot to the adjacent spot and the eventual contamination of the needle tip are also expected. Third, except for thin sliced sections, real samples are not flat, and therefore, the needle sampling depth could vary throughout the raster scanning process. To circumvent these problems, Chen *et al.* added an auxiliary solvent vapor sprayer to supply uncharged solvents (aqueous, or water/acetonitrile) to the probe tip when the needle was at the ionization position.^30)^ The probe could be wetted by the condensation of solvent vapor. In addition, to maintain a constant sampling depth into the sample surface, an automatic control of the sample stage in the vertical *z* axis was also implemented in addition to the raster *x*–*y* scanning. The proof-of-principle of imaging PESI/MS was performed on a mouse brain section, the lipid components of which have been well studied by imaging MALDI/MS. The distribution of phosphatidylcholin (PC) and galactosylceramides (GalCer) in the region of white and grey matter agreed reasonably with those obtained by MALDI-MS. However, while the mapping of PC and GaLCer needs to be conducted separately using different MALDI matrices,^37,38)^ both lipids could be readily detected and mapped by PESI. The lateral resolution was ∼60 μm, and higher resolution is possible by downsizing the needle probe (*e.g.*, use of aluminum-coated glass tip manufactured for scanning near-field optical microscopy with a tip diameter of 10 s of nm).

### Application to single cells

Single cells are the minimal units of living organisms. Most of the cellular chemical/biological research studies are performed on cell populations, which contain a number of cells due to the assumption that cells from the sample type are identical in both chemical components and biological behavior. However, more experimental evidence has shown the cell-to-cell variability within the same cell population. Conventional cell research methods using population level cells for analysis can obscure the cell-to-cell heterogeneity and cause misunderstanding of functions of the cell population. Thus, it is of great importance to develop methods for single cell analysis to disclose the cell-to-cell differences, which will be helpful to understand environmental stress on the cells.

**Figure 5:**
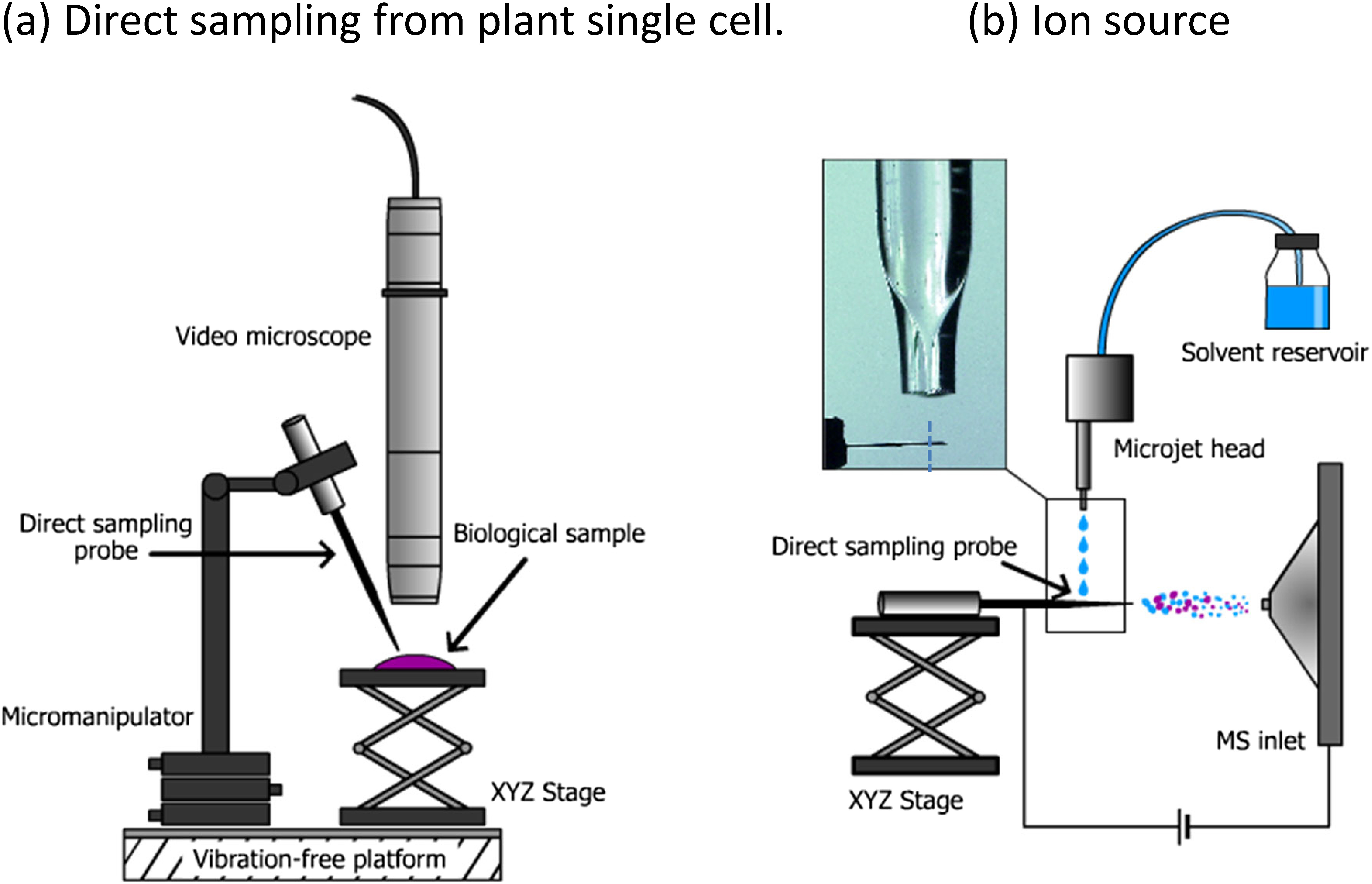
Fig. 5. Schematic diagram of the instrumental setup for the single cell observation. (a) Direct sampling from plant single cells, and (b) the ion source. Reproduced by permission of The Royal Society of Chemistry.

**Figure 6:**
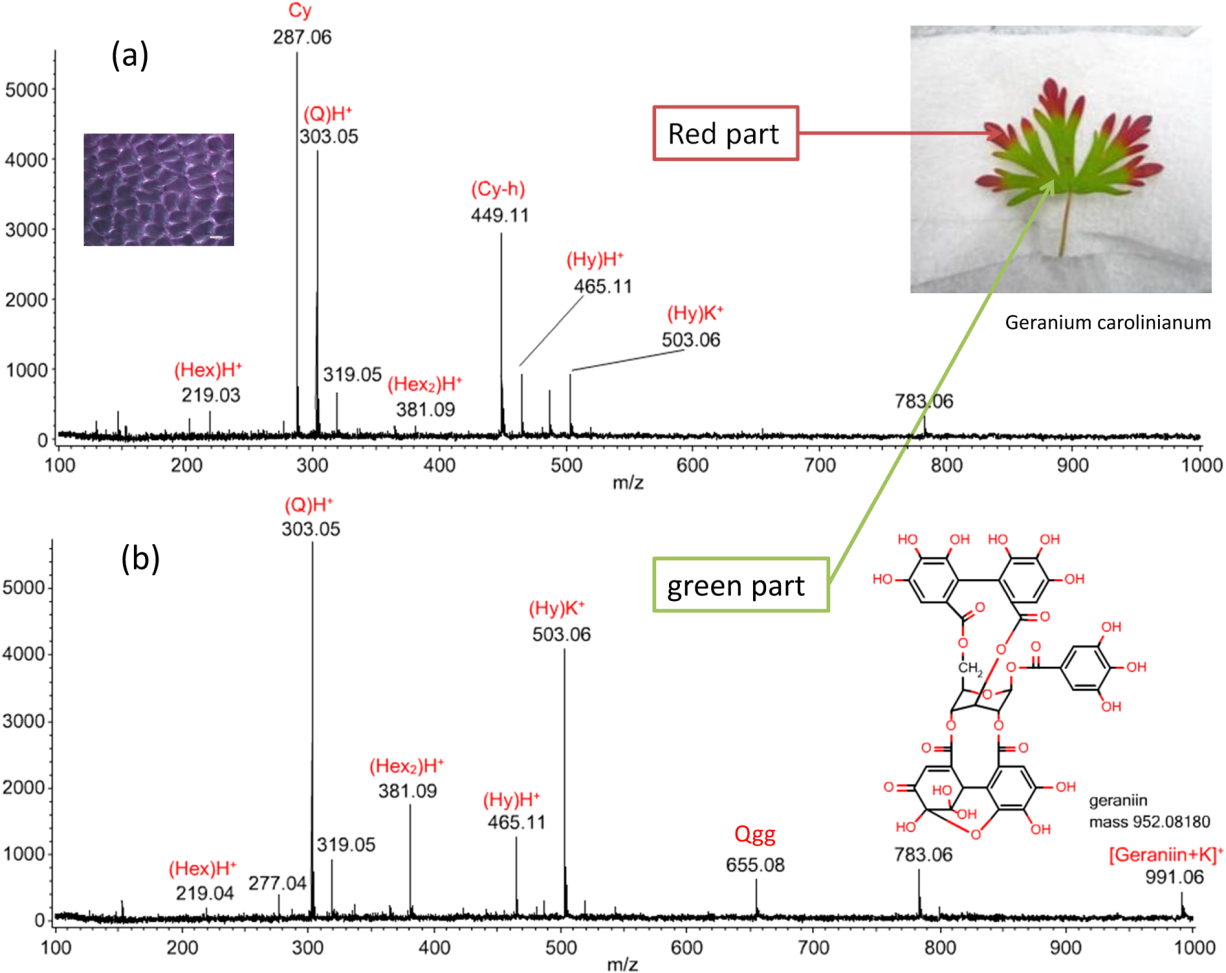
Fig. 6. Direct profiling of metabolites in plant cells. (a) Red part of a *Geranium carolinianum* leaf. (b) Green part of a *Geranium carolinianum* leaf. Cy: cyanidin, Hy: hyperocide, Q: quercetin, Qgg: quercetin-3-*O*-β-D-(6′-galloyl) galactosidase, h: hexoside. Reproduced by permission of The Royal Society of Chemistry.

Yu *et al.* presented modified PESI consisting of a stainless steel sampling probe (body diameter: ∼30 μm) with sufficient sharpness (tip diameter of ∼100 s of nm), high surface hydrophilicity and a piezoelectric inkjet system as the solvent microdroplet (∼30 μm in diameter) provider.^39)^ A schematic diagram of the instrumentation is depicted in Fig. 5. A piezoelectric ink jet system (IJK-200HS, Microjet Inc., Tokyo, Japan) equipped with an ink jet head (AD-K-501) was used as the solvent droplet generator. With a piezoelectric frequency of 50 Hz, the solvent (water/acetonitrile) was supplied to the needle with the flow rate of ∼40 nL min^−1^. By penetrating the single cell, the sampling probe was estimated to load less than 1 pL biofluid from an individual cell. Figure 6 shows the mass spectra of single cells from the red part and green part of *Geranium carolinianum* leaves. Saccharides and flavonoids including quercetin (Q) and hyperocide (Hy) were observed in both the red-senescing and green parts. Due to the senescence effect induced accumulation of anthocyanins, the red-senescing part gave strong signals of cyanidin (Cy) and its glycosides. The most interesting result is the detection of geraniin in the cells from the green part but not from the red-senescing part. This shows that the geraniin content varies with the leaf age. The key point for the success of this study was to make the needle surface superhydrophilic by etching the needle in 30% (w/w) H_2_SO_4_ solution containing KMnO_4_ and K_2_Cr_2_O_7_.

## DIPPING PESI (dPESI)

**Figure 7:**
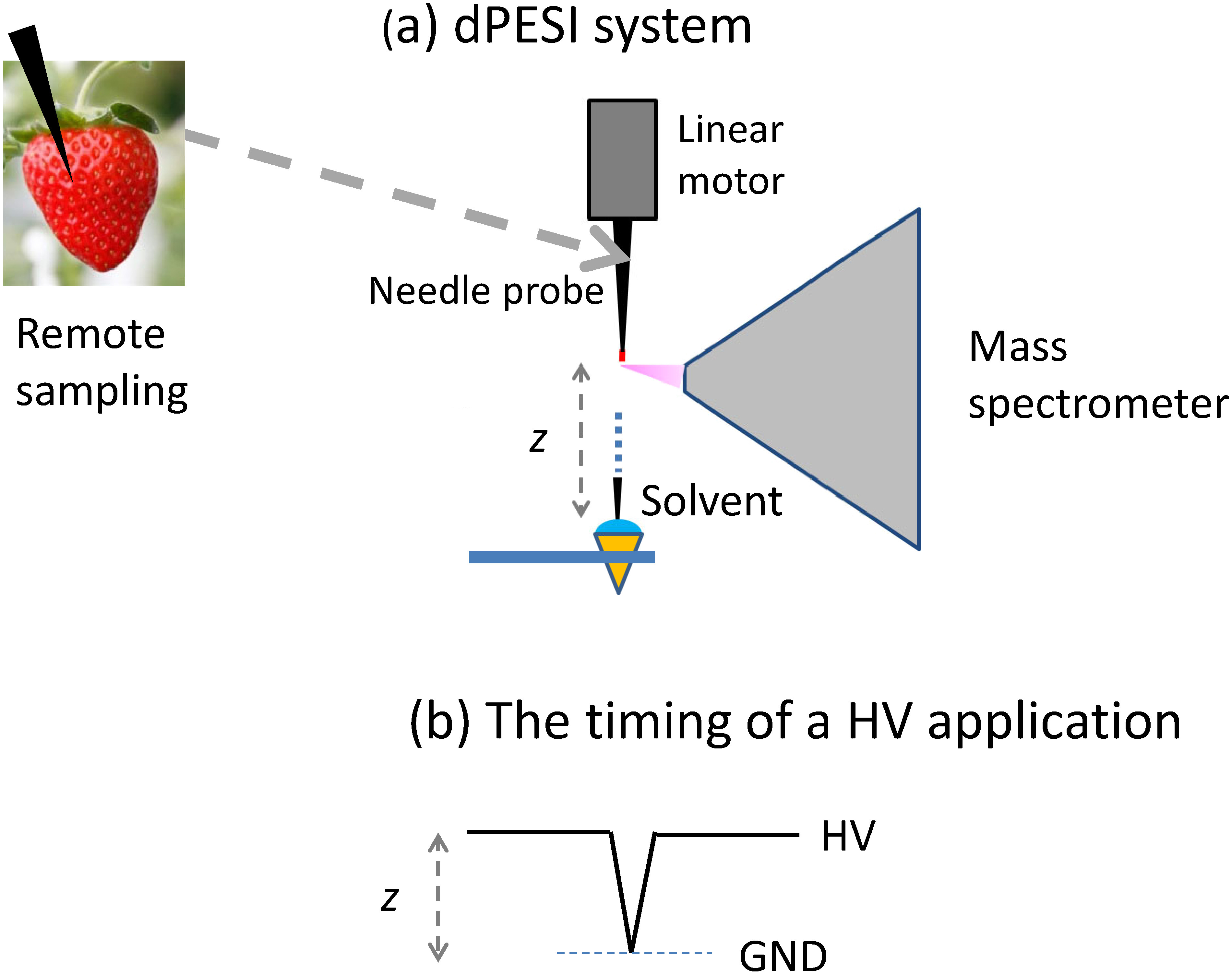
Fig. 7. (a) Schematic diagram of the dPESI system. The needle tip bearing dried sample was dipped into the pure solvent to a depth of ≤1 mm. (b) The timing of the probe operation and application of a high voltage to the needle is synchronized. The high voltage was applied to the needle at its highest position. Reproduced from Ref. 14, copyright 2018, with permission from Elsevier.

The use of vapor condensation^30,31)^ for supplying solvent to the needle requires sophisticated skills in order to achieve reproducible results. To avoid the operational complexity, a much simpler method for supplying solvent to the needle, dipping PESI (dPESI), was developed.^14)^ Figure 7 shows the schematic diagram of the dPESI system. Sampling is performed by pushing the acupuncture needle into the sample to a depth of ∼0.5 mm, just like an acupuncture treatment. After drying the sample, the needle is positioned in front of the mass spectrometer. The needle is then moved down and wetted by just dipping it for ∼50 ms into the pure liquid solvent, and then lifted. At the highest position, the liquid sample is electrosprayed by applying a HV to the metal needle. This operation is exactly the same as in conventional PESI, except that the sample is *preloaded* at the needle tip and that the needle is dipped into *pure* solvent. Because the sampling by a needle and the mass spectrometric measurement are performed independently, dPESI is applicable to any real-world bulky samples for a low-invasive direct analysis of any types of samples, *e.g.*, plants, meats, biological tissues, and foods.

**Figure 8:**
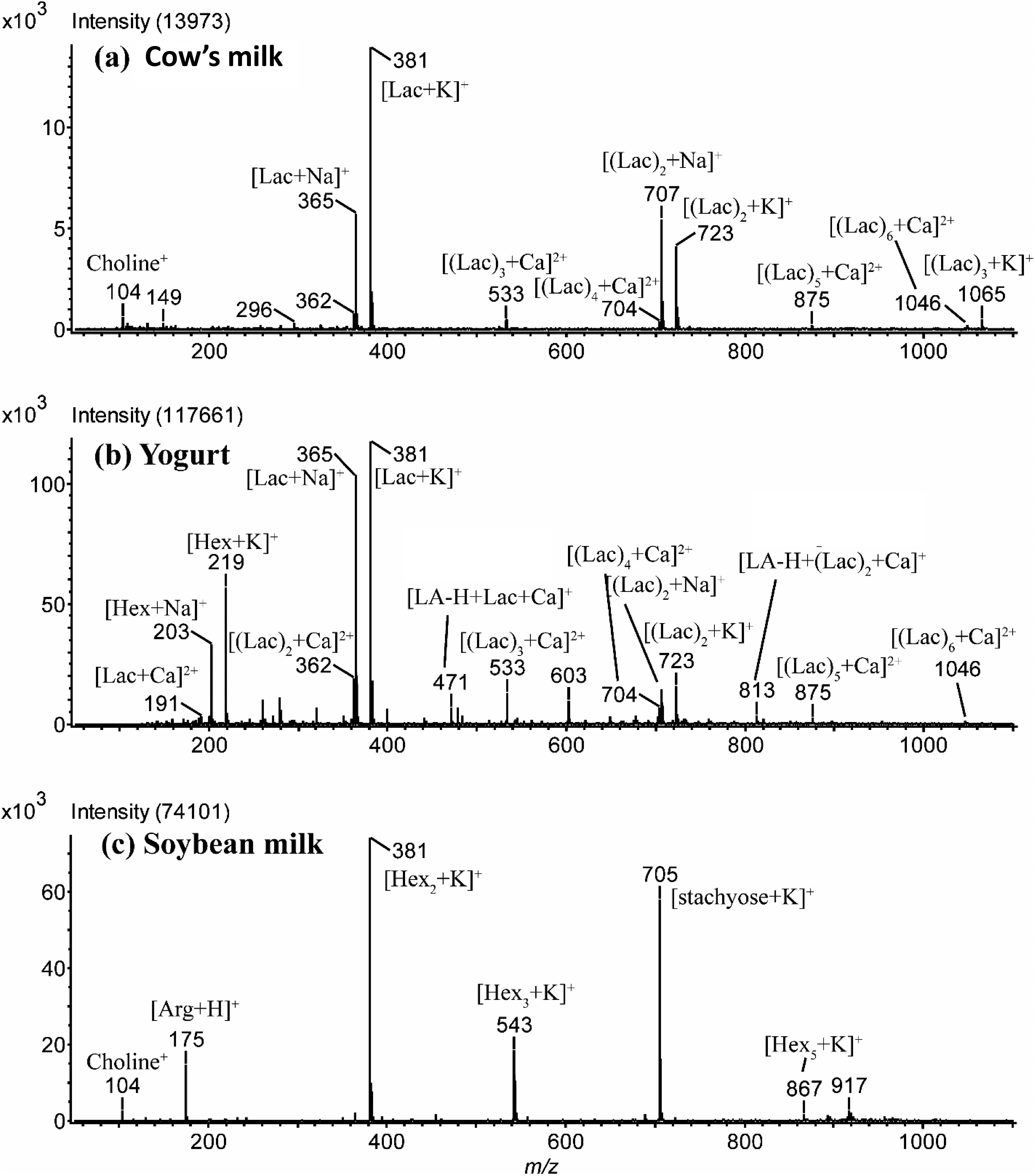
Fig. 8. Mass spectra for (a) cow’s milk, (b) yogurt, and (c) soybean milk. Lac stands for lactose and LA stands for lactic acid. Hex, Hex_2_, and Hex_3_ stand for monosaccharide, disaccharide, and trisaccharide, respectively. Reproduced from Ref. 14, copyright 2018, with permission from Elsevier.

**Figure 9:**
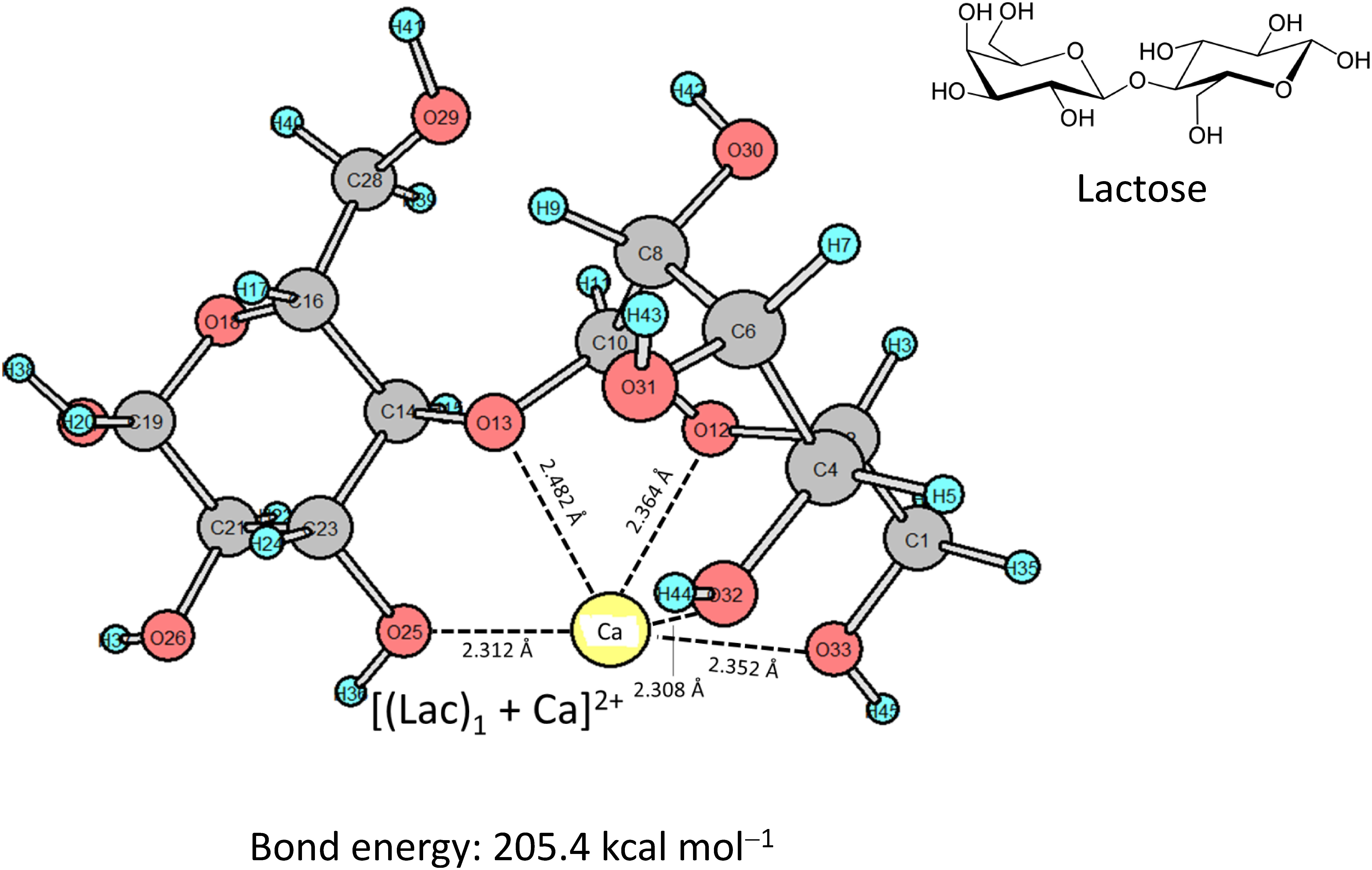
Fig. 9. Geometry of [Lac+Ca]^2+^ optimized by DFT calculations. Lac: lactose. Reproduced from Ref. 14, copyright 2018, with permission from Elsevier.

As an example, mass spectra for cow’s milk, yogurt, and soybean milk are shown in Fig. 8.^14)^ Figures 8(a) and (b) show the mass spectra for cow’s milk and yogurt, respectively. The major ions detected in these two mass spectra were assigned as the adduct ions of Na^+^, K^+^, and Ca^2+^ with lactose (Lac) molecules. Studies of metal ion solvation provide information not only about metal ion chemistry in solution, but also can lead to an improved understanding of the structure and functions of many biomolecules in which metal ion interactions play a role. The multiply charged metal ions are more difficult to detect than the singly charged ions because of their higher solvation energies. Dairy foods are a major source for the intake of calcium. However, lactose intolerance often results in adverse dietary modifications. Thus, more studies are needed to understand lactose intolerance and how it relates to calcium intake and various health conditions. The appearance of [(Lac)*_n_*+Ca]^2+^ for milk (*n*=3–6) and yogurt (*n*=1–6) in Figs. 8(a) and (b) suggests that the ligand Lac molecules form the strong bonds with the core ion of Ca^2+^. The interacting systems of Ca^2+^ with Lac molecules were investigated by density functional theory (DFT) calculations. The geometry of the Ca^2+^⋅⋅⋅⋅Lac is shown in Fig. 9.^14)^ Its estimated bond energy (205.4 kcal mol^−1^) is extremely large. The strongly bound cluster ions of Ca^2+^ with Lac molecules in dairy foods might play an important regulatory role in a number of specialized functions in the body. In the mass spectrum for soybean milk (Fig. 8(c)), potassiated stachyose, the main component of oligosaccharides in soybean, was detected as one of the major ions.

dPESI was applied to many other samples, *e.g.*, plants, fish, animal tissues, and highly viscous fermented soybeans (Natto) without any sample pretreatment.^14)^ They gave characteristic mass spectra with strong ion intensities. It should be noted that by changing the solvents, different kinds of components could be preferentially extracted depending on their hydrophobicities.

## SHEATH-FLOW PESI (sfPESI)

**Figure 10:**
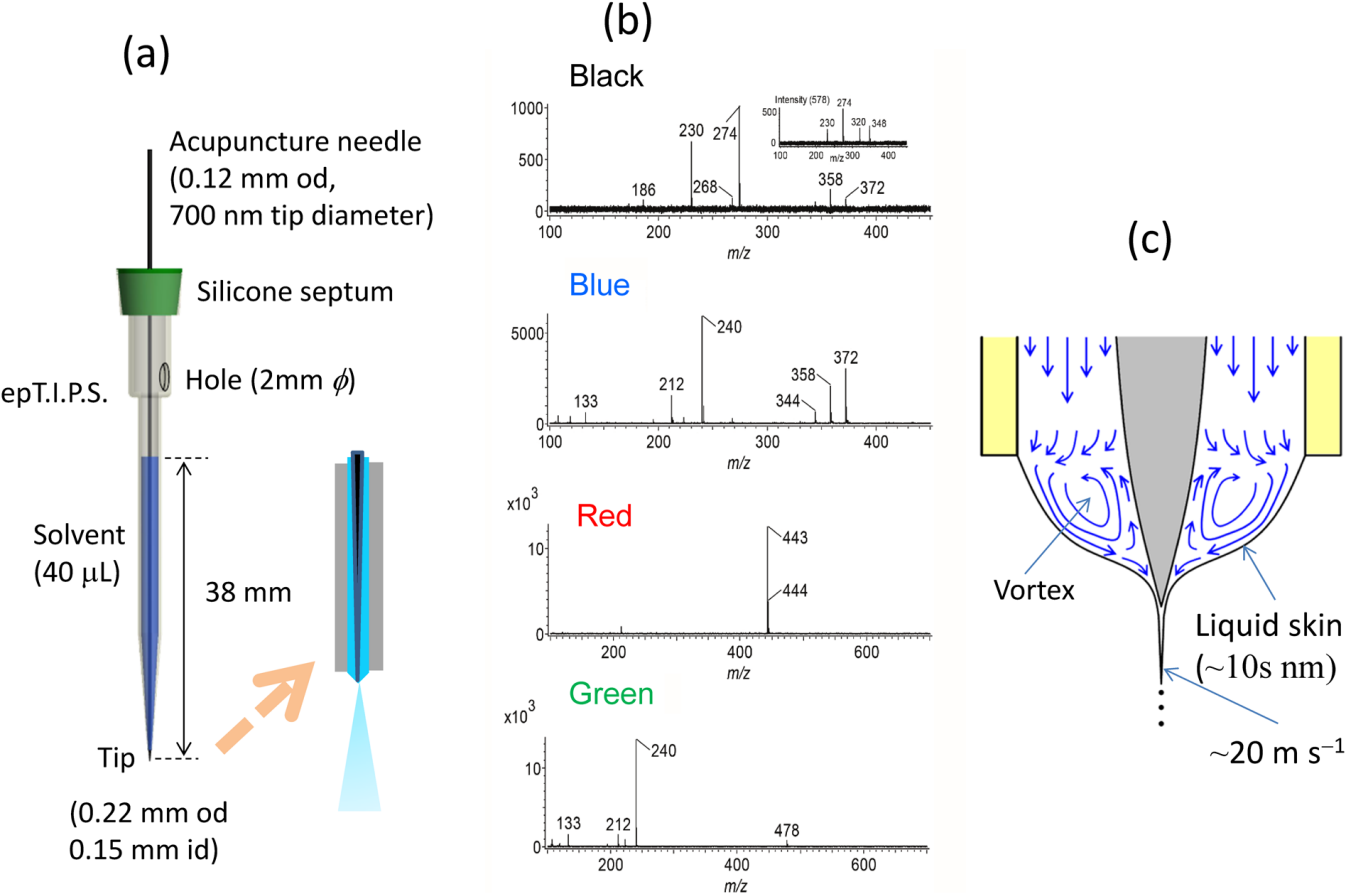
Fig. 10. (a) Structure of the probe used on sfPESI. An acupuncture needle (body diameter: 0.12 mm, tip diameter: 700 nm) was inserted into the gel-loading tip (epT.I.P.S., 20 μL, Eppendorf) with a protrusion of 0.1 mm. The metal needle was fixed by using a silicone septum. Solvent as added in the capillary through a hole opened at the upper tapered part of the capillary. The solvent liquid head was 38 mm. (b) Mass spectra for lines of a four-color ballpoint pen drawn on the paper. (c) Vortex formed in the Taylor cone. Reproduced from Ref. 15, copyright 2018, with permission John Wiley and Sons.

As described above, sampling and electrospray in dPESI were performed independently and these operations can be carried out under optimized conditions. If these two operations can be unified, the experimental systems would be greatly simplified. To fulfill this idea, sheath-flow PESI (sfPESI) was developed.^15)^ The probe for sfPESI is shown in Fig. 10(a). An acupuncture needle (body diameter: 0.12 mm, tip diameter: ∼700 nm) is inserted into a gel-loading tip. The solvent for sample extraction is filled through a 2-mm-diameter hole located in the upper part of the gel-loading tip with a liquid head of ∼38 mm. The total amount of solvent loaded in the capillary is 40 μL. The outer and inner diameters of the probe tip were 0.22 and 0.15 mm, respectively. The solvent used for sample extraction was water/methanol (1/1) or water/methanol/acetonitrile (1/1/1). By coating the outer surface of the gel loading tip with perfluoroalkyl film (FG-5093SH-0.5, Fluorotechnology, Kasugai, Japan) and causing the needle to protrude by 0.1–0.2 mm from the tip of the plastic capillary, stable single cone electrospray along the axis of the capillary is generated. Figure 10(b) shows the mass spectra obtained for a four-color (black, blue, red and green) ballpoint pen (Zebra, Clip-on, Multi). No analyte ions were detected when water/methanol (1/1) was used for the solvent, because ballpoint pen inks were nearly insoluble in this solvent. In contrast, when the solvent was changed to water/methanol/acetonitrile (1/1/1), strong ion signals appeared (Fig. 10(b)). Apparently, acetonitrile was effective in extracting analytes in oleaginous ballpoint pen inks. It is evident that sfPESI techniques bring substantial simplification over existing mass spectrometric ambient approaches that employ a free-standing liquid junction between the capillary and the sample surface.

sfPESI has been applied to various samples such as coffee powder grains, tablets, rice grains, and animal samples.^15)^ In many cases, sequential electrospray was observed as in the case of PESI. In PESI and also in sfPESI, electrospray was generated by spontaneous self-aspiration. Surface components of samples are extracted into the solvent with a solvent volume of about 2 nL at the capillary tip.^15)^ The electrospray of the analytes lasted ∼3 s, during which about 40 nL solvent was expelled from the capillary with a solvent flow rate of ∼800 nL min^−1^. The residence time for 2 nL solvent in the capillary was only ∼0.2 s. The duration of analyte ion signals (∼3 s) was much longer, meaning that the analytes were sparingly electrosprayed in sfPESI. This phenomenon may be explained by the liquid circulation in the Taylor cone. Hayati *et al.* observed the axisymmetric circulating meridional motion of the tracer particles (lycopodium powder) in the conical base of the Taylor cone.^40)^ The velocity of the liquid at the surface layer was the largest, whereas there was a backflow at the center. This represented almost the opposite of the flow velocity distribution for the laminar flow in a tube where the velocity is zero at the wall of the tube and maximum at the center. This liquid circulation (*i.e.*, vortex) is driven by the surface shear stress induced by the tangential electric field. The thickness of the surface layer enriched by the excess charges is of the order of Debye length (λ_D_)^25)^ (∼10 s of nm) and only the surface skin is ejected through the jet while the rest recirculates along the axis.
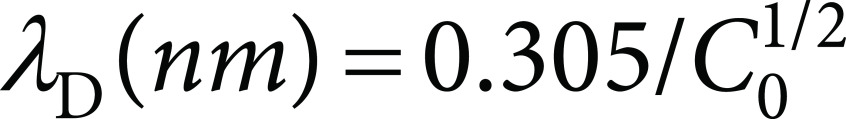
(7)

The axis of the donut-shaped toroidal vortex coincides with the centerline of the cone (Fig. 10(c)). It is likely that surface-active ions are preferentially enriched in the thin liquid surface skin, whereas less surface-active ions are temporarily preserved in the stagnation region of the vortex. That is, the long duration of electrospray of analytes and the occurrence of the sequential electrospray must be closely related to the toroidal vortex in the liquid cone.

**Figure 11:**
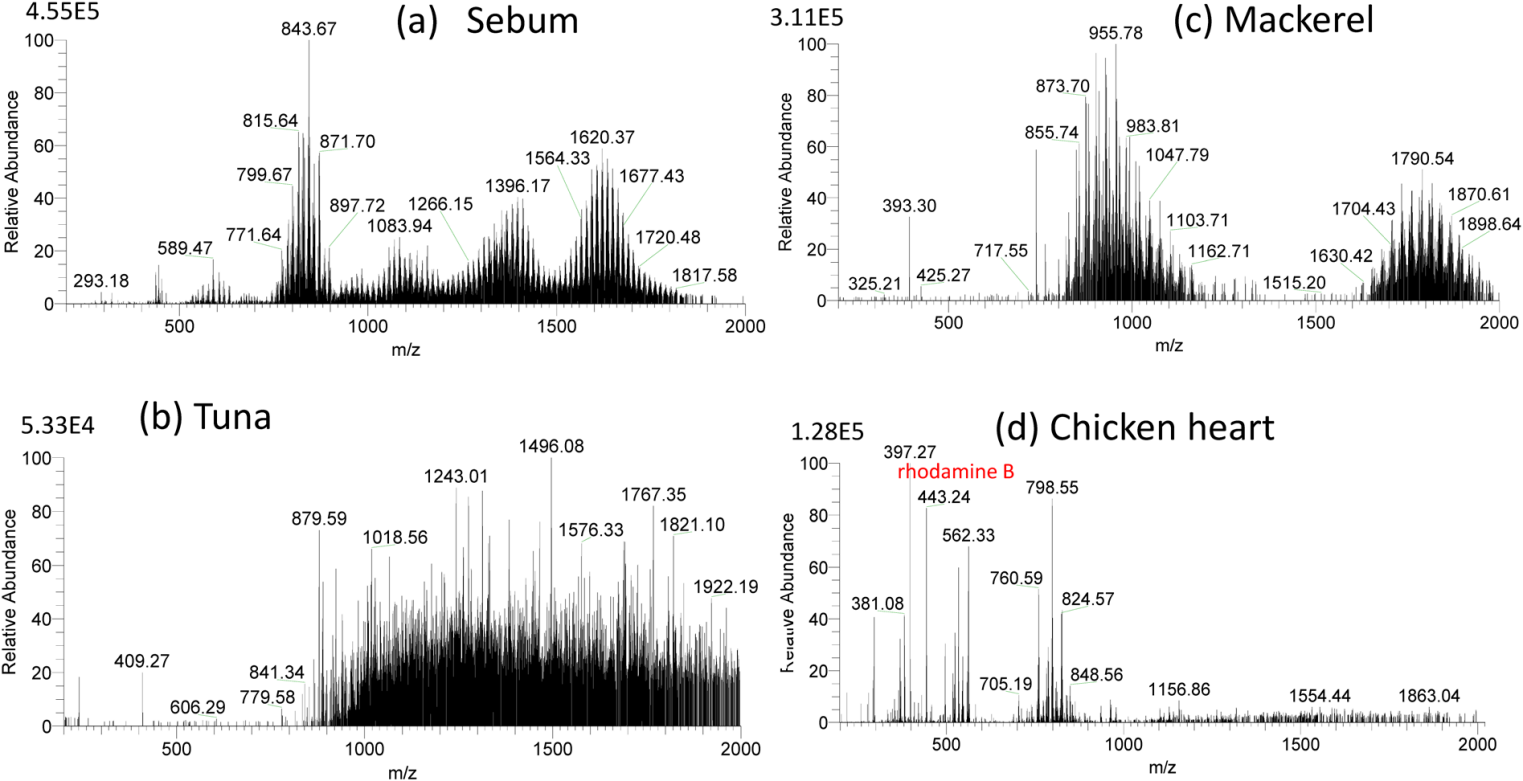
Fig. 11. sfPESI mass spectra for (a) sebum taken from the forehead of a volunteer, (b) sliced tuna meat (sashimi), (c) mackerel meat, and (d) chicken heart purchased from a supermarket. Rhodamine B (red dye) may be the artificial coloring matter. Reprinted with permission from Ref. 46. Copyright (2020) American Chemical Society.

Figures 11(a)–(d) show the sfPESI mass spectra for sebum taken from the forehead of a volunteer, sliced tuna, mackerel, and chicken heart, respectively.^46)^ Rhodamine B detected in Fig. 11(d) may be the artificial coloring matter added in commercial chicken heart.

## ADJUSTABLE sfPESI (ad-sfPESI)

Land plants are coated by epicuticular wax that covers the surface of the plant cuticles, and primarily consists of hydrophobic organic compounds. The main function of the epicuticular wax is to decrease surface wetting and moisture loss. Because of the nonwetting properties of the plant surface, sfPESI is in some cases difficult to apply to plants. To solve this problem, adjustable sfPESI (ad-sfPESI) was developed.^16)^ In ad-sfPESI, exactly the same probe utilized in PESI is used. The only difference is that the sample is punctured by the acupuncture needle that is protruding from the tip of the capillary by 5 mm. After pricking the sample surface with an invasion depth of ∼1 mm, the needle is retracted into the capillary with a protrusion length of 0.1∼0.2 mm. Because the sampling is conducted remotely from the mass spectrometer, on-site and *in situ* sampling is straightforward as in the case of dPESI. Ad-sfPESI has been applied to a variety of plants such as fruits and vegetables. Characteristic mass spectra were obtained for *all* the samples tested without clogging of the capillary. This is due to the fact that samples extracted from plants are basically water-soluble and almost all the components are washed out by solvent without leaving any residues in the capillary. Because the body diameter of the acupuncture needle used was 0.12 mm, a site-specific point analysis with the spot diameter of ≤0.2 mm can be achieved.

**Figure 12:**
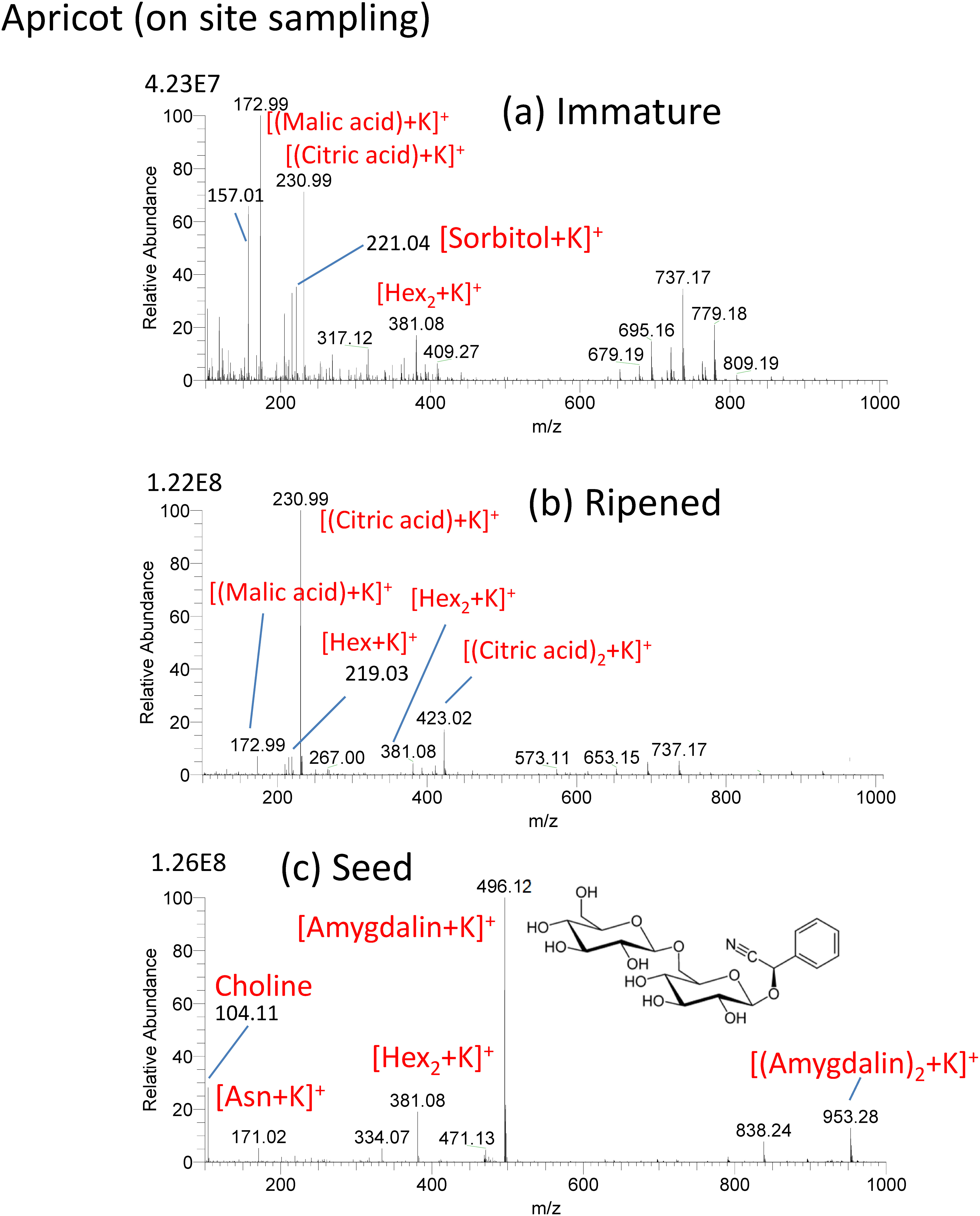
Fig. 12. Mass spectra for Japanese apricot obtained by on-site sampling. (a) Immature green apricot measured on May 5th, 2018. (b) Ripened Japanese apricot measured on June 12th, 2018. (c) Seed taken out from the ripened apricot. Reprinted with permission from Ref. 16. Copyright (2019) American Chemical Society.

*In situ* analysis of the developing stage of Japanese apricot (Bungo plum) was made by ad-sfPESI.^16)^ Figure 12(a) shows the mass spectrum for a young hard green apricot growing in the garden sampled *in situ* on May 5th, 2018. After sampling, the probe was brought back to the laboratory and ad-sfPESI mass spectrum was measured. [(malic acid)+K]^+^ (*m*/*z* 172.99), [(citric acid)+K]^+^ (*m*/*z* 230.99) and [sorbitol+K]^+^ (*m*/*z* 221.0) were detected as major ions. Figure 12(b) shows the mass spectrum for the same fruit as that shown in Fig. 12(a) after full ripening on June 12th, 2018. [(citric acid)+K]^+^ became the major ion, and the ion intensity of [(malic acid)+K]^+^ was much weaker than that of [(citric acid)+K]^+^. It should be noted that the ratios of intensities of [sorbitol+K]^+^ (*m*/*z* 221.04) to [Hex+K]^+^ (*m*/*z* 219.03), [sorbitol+K]^+^/ [Hex+K]^+^, are 10.1 for the immature apricot and 0.26 for the ripened apricot (Hex: hexose). In Rosacease plants such as apricot, the assimilatory starch photosynthesized in leaves is translocated to fruits as sorbitol and sucrose *via* the phloem. The translocated sorbitol in fruits is then converted to saccharides (fructose, glucose, sucrose, *etc.*). The observed drastic change of the ratio [sorbitol+K]^+^/[Hex+K]^+^ from 10.1 for immature and 0.26 for ripened apricot indicates that almost all the sorbitol is converted to Hex in the ripening stage. The stronger [Hex_2_+K]^+^ (1.27E7) than [Hex+K]^+^ (2.77E6) in the immature apricot may be attributed to the fact that the assimilatory starch is translocated as Hex_2_ as well as sorbitol. Figure 12(c) shows the mass spectrum for the seed taken from the ripened apricot shown in Fig. 12(b). Potassiated amygdalin [amygdalin+K]^+^ (*m*/*z* 496.12) was detected as the major ion. Amygdalin is a family of hydrocyanic acid glycosides and is known to be toxic because it produces hydrocyanic acid by the action of β-glycosidase.

**Figure 13:**
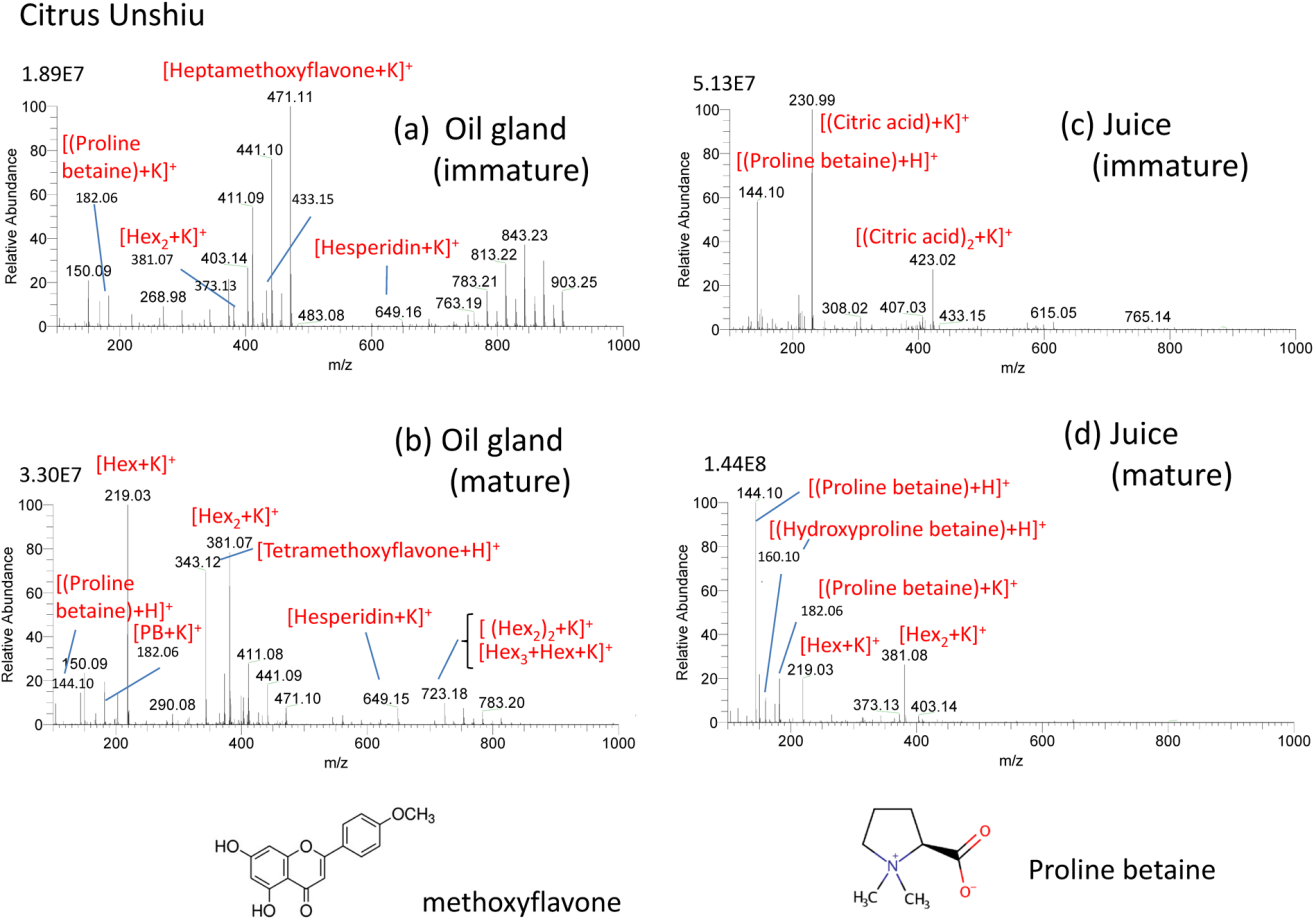
Fig. 13. Mass spectra for (a) an oil gland from a green immature orange peel, (b) an oil gland from a fully ripened orange peel, (c) juice of a premature orange, and (d) juice of a fully ripened orange. The immature orange was harvested on July 21st, 2018, and the ripened one was harvested on October 10th, 2018. PB stands for proline betaine. Reprinted with permission from Ref. 16. Copyright (2019) American Chemical Society.

Plants are unable to move and they must be able to alter their growth in such a way that is best suited to climate, season and their environment. Yu *et al.*^41)^ and Liu *et al.*^42)^ pointed out that components of phytochemicals in plants change dramatically with the parts and development stage. Herein, ad-sfPESI was applied to the direct analysis/profiling of bioactive compounds and the monitoring of metabolic changes in orange (*Citrus unshiu*).^16)^ Because the acupuncture needle with a body diameter of 0.12 mm was used, the point analysis with the diameter of ≤0.2 mm was feasible. To demonstrate this advantage, an oil gland of an orange peel with a diameter of ∼0.5 mm was measured in different seasons to examine the component change for the developing stage. Figures 13(a) and (b) show the mass spectra for oil glands of immature and mature orange. In Fig. 13(a) for the immature orange harvested on July 21st, 2018, protonated polymethoxyflavones ([PMF+H]^+^) in the *m*/*z* region of 343∼471 were detected. Tetra-, penta-, hexa-, and heptaMF were the major components in the immature orange. The group of ions in the range of *m*/*z* 700–900 are attributed to potassiated mixed clusters of polymethoxyflavones. Flavonoids protect the plants from ultraviolet light and also play a role of antibacterial action. Rich PMF in immature orange peel is generally observed for citrus as reported by Hyun *et al.*^43)^ Ji *et al.* also reported that PMFs exist exclusively in the citrus, particularly in the peel of sweet orange.^9)^ Figure 13(b) shows the mass spectrum for an oil gland of the mature orange harvested on October 10th from the same orange plant in the garden. [Hex+K]^+^ (*m*/*z* 219.03) that was barely observed in Fig. 13(a) became the base peak. Among PMFs, [tetraMF+H]^+^ (*m*/*z* 343.12) was detected as the strongest peak in agreement with the previous work.^9)^ PMFs have attracted attention as bioactive compounds in Citrus peels owing to their anti-inflammatory, anti-carcinogenic, anti-atherogenic and hepatoprotective effects.^42,44)^ Proline betaine detected in Figs. 13(a) and (b) is known as a biomarker of citrus, and it functions as a highly effective osmoprotectant that accumulates in a variety of plant species in response to environmental stresses such as drought, salinity, extreme temperature, UV radiation, and heavy metals. While [(citric acid) +K]^+^ was detected as the major ion for immature orange in Fig. 13(c), it was barely detected for mature orange in Fig. 13(d).

## COMPARISON OF PESI, dPESI, sfPESI AND ad-sfPESI

**Figure 14:**
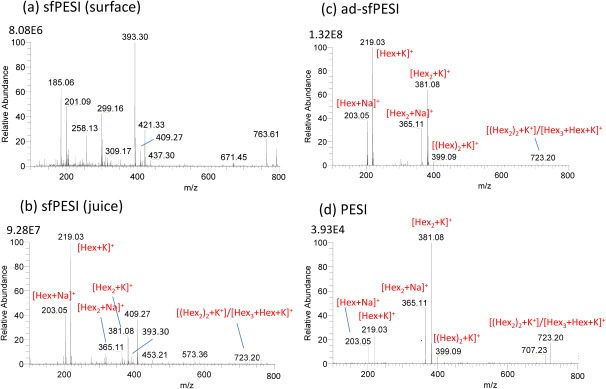
Fig. 14. (a) sfPESI mass spectrum for the skin of the apple. (b) sfPESI mass spectrum for the apple flesh. (c) ad-sfPESI mass spectrum for the apple. (d) PESI mass spectrum for the apple flesh. The peak at *m*/*z* 707.23 was not identified. Hex, (Hex)_2_, Hex_2_, and Hex_3_ stand for monosaccharide, monosaccharide dimer, disaccharide, and trisaccharide, respectively. Reprinted with permission from Ref. 16. Copyright (2019) American Chemical Society.

For the comparative study of PESI, sfPESI and ad-sfPESI, an apple was analyzed by these three methods.^16)^ Figure 14(a) shows the sfPESI mass spectrum measured by touching the probe tip to the peel of the apple. The liquid meniscus at the probe tip was made in contact with the peel, but the surface was not wetted by the solvent, *i.e.*, the liquid was repelled from the surface. The ions at *m*/*z* 393.30, 409.27 and 763.61 originated from methanol used as a solvent (water/methanol (1/1)). Numerous peaks detected in Fig. 14(a) were not identified.

Agricultural chemicals typically used in apple production, such as acetamiprid, kresoxim-methyl, diflubenzuron, cyfluthrin, trifloxystrobin, and fenpropathrin, could not be detected. Furthermore, saccharides, which are a major component of apples, were also not detected. Based on this, it is apparent that the wax surface of the apple acts as a barrier for water-soluble compounds. Figure 14(b) shows a mass spectrum from the analysis of apple flesh by sfPESI. In this experiment, the peel of the apple was removed and the sfPESI probe was touched to the apple flesh. The major ions detected were sodiated and potassiated Hex and Hex_2_, and the contaminating solvent ions detected in Fig. 14(a) were not detected. Figure 14(c) shows the mass spectrum obtained using ad-sfPESI. In this experiment, the needle protruding from the end of the gel loading tip was used to directly pierce the apple skin. A similar mass spectrum to that obtained by sfPESI was observed, with sodiated and potassiated saccharides detected as the major ions. This demonstrates the major advantage of ad-sfPESI: the ability to bypass the interference of the outer sample surface and enable analyte collection. Upon retraction of the needle into the capillary, the collected sample liquid replaces the solvent in the narrow channel between the inner wall of the capillary and the needle. This may be why the mass spectrum obtained by ad-sfPESI is free from contaminants present in the solvent and on the surface of the sample. In ad-sfPESI, solvent extraction of analytes does not occur. This is the primary difference between ad-sfPESI and sfPESI. Finally, Fig. 14(d) shows the mass spectrum obtained using conventional PESI in the analysis of apple flesh. In this experiment, a thin slice of apple flesh was punctured by the PESI needle to a depth of ∼0.5 mm. Sodiated and potassiated saccharides were once again detected as with the use of sfPESI and ad-sfPESI, though with a notable difference in the relative abundance of these compounds. In the use of all PESI-related techniques, it was found that the mass spectra were typically site-specific, with differences in mass spectra observed depending on the sampling point. Nakashima *et al.* previously demonstrated the striking difference in the metabolic composition of two adjacent cells types of tomato trichomes by PESI analysis, further highlighting the importance of sampling site.^45)^

dPESI,^14)^ sfPESI,^15)^ and ad-sfPESI^16)^ are originated from PESI.^8,11)^ These methods have their own advantages and disadvantages depending on their methodological principles. Remote sampling away from the mass spectrometer is feasible by dPESI, sfPESI and ad-sfPESI. Both PESI and dPESI are applicable to the analysis of liquid samples and viscous materials such as plant and animal tissues. dPESI can furthermore be applied to the analysis of dry samples, however additional sample preparation is required to first wet the material with a solvent. sfPESI is widely applicable to a range of sample types, including liquid, dry and viscous samples, making it the most versatile of the PESI-derived techniques. Finally, ad-sfPESI is applicable to the analysis of plants, however is not suitable for the analysis of viscous samples such as animal tissues due to problems of capillary clogging. dPESI, sfPESI and ad-sfPESI are additionally suitable for remote sampling, enabling the *in situ* sampling of a material prior to transportation to the laboratory for analysis.

Table 1 together with the conceptual idea of PESI, dPESI, sfPESI and ad-sfPESI summarizes the applicability of the four methods under various experimental conditions.^46)^

**Table 1:** Table 1. Applicability of PESI, dPESI, sfPESI and ad-sfPESI together with their schematic diagrams.

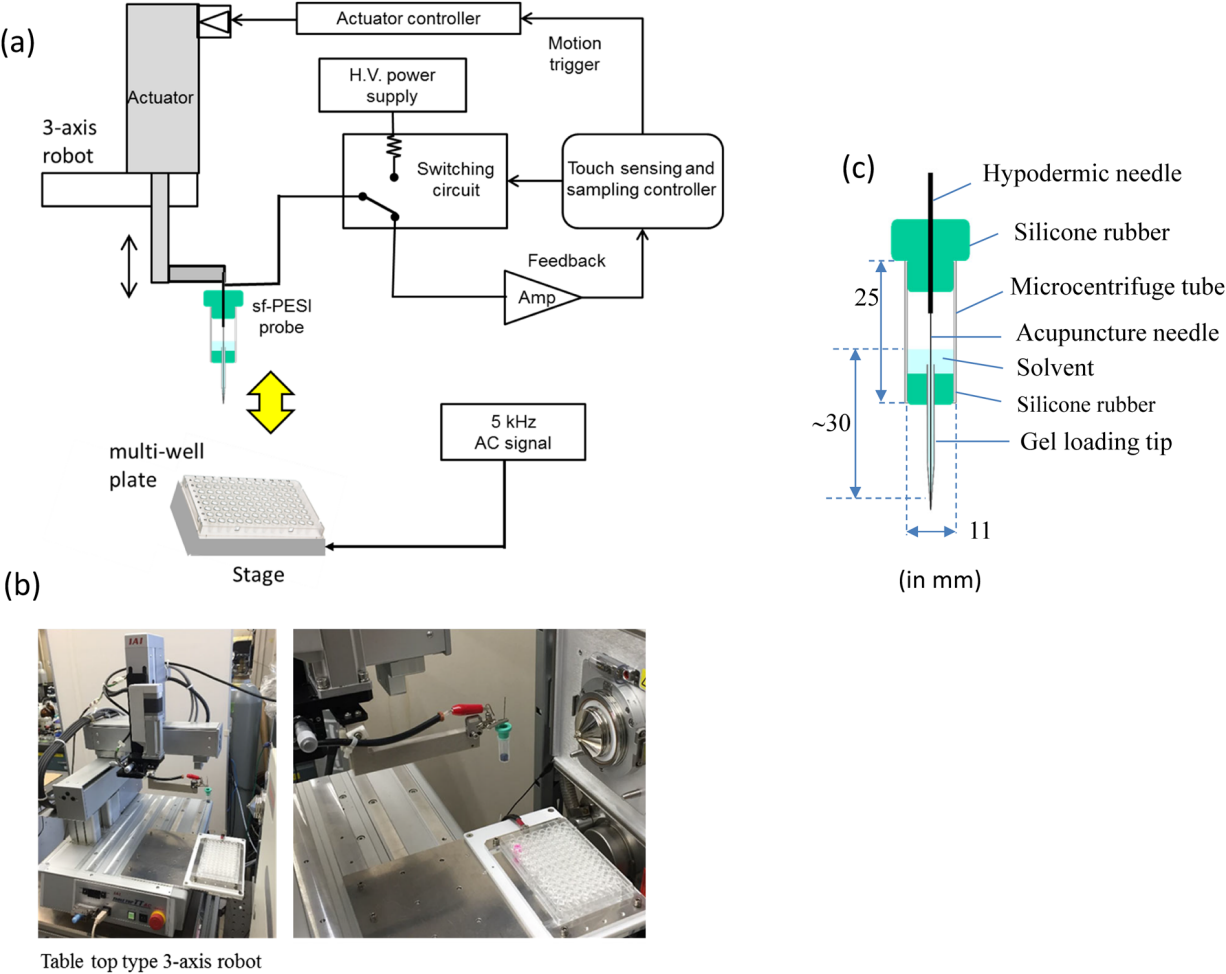

○: readily applicable, △: applicable but some sample preparation is necessary, ×: not applicable. Reprinted with permission from Ref. 46. Copyright (2020) American Chemical Society.

## ROBOTIC sfPESI/MS EQUIPPED WITH A TOUCH SENSOR

**Figure 15:**
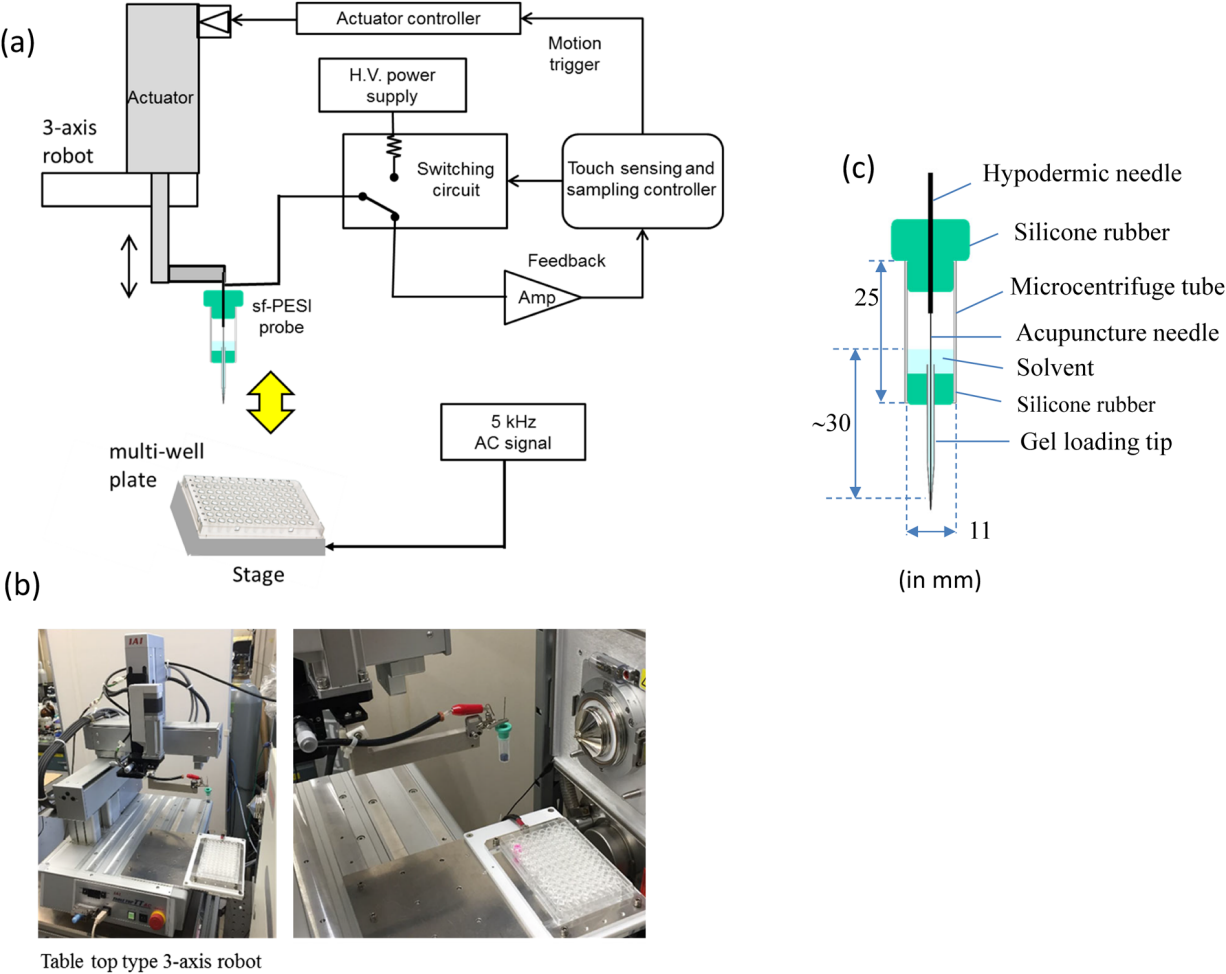
Fig. 15. (a) Schematic diagram of the electrical circuit of the touch sensor for sfPESI. The voltage and frequency applied to the stage were 5 kHz and 4.5 V_pp_, respectively. (b) The linear actuator fixed to an AC servo motor tabletop 3-axis robot (IAI, TTA-C3SH-WA-20-15-15B). (c) sfPESI probe used for sampling and ionization. Water/methanol (1/1) solvent was used for the extraction of samples. About 1 mL solvent was prefilled in the reservoir with the liquid head of ∼30 mm. The outer surface of the gel loading tip was coated with a perfluoroalkyl film to make the surface hydrophobic. Unit for the number: mm. Reproduced by permission of The Royal Society of Chemistry.

As shown in Table 1, sfPESI is the most versatile method for surface analysis while others are better suited to bulk analysis. If a touch sensor is installed for sfPESI, it would be very promising for surface analysis of samples which are located remote from the mass spectrometer (*i.e.*, remote-sampling mass spectrometry). For the quick, noninvasive, and high-sensitivity surface analysis of liquid and solid materials, a touch sensor for sfPESI was developed and coupled with a 3-axis robotic system.^46)^ A schematic diagram of the electrical circuit of the touch sensor is shown in Fig. 15(a).^47)^ The capacitance between the probe and the sample placed on the metal stage was monitored by the circuit during the sampling motion. By detecting the sudden increase in the displacement current flowing through the circuit at the contact point, the programmable linear actuator stops at that position. The optimized voltage and frequency applied to the stage were 5 kHz and 4.5 V_pp_, respectively, for the highest sensitivity. The linear actuator was fixed to an AC servo motor tabletop 3-axis robot (IAI, TTA-C3SH-WA-20-15-15B) as shown in Fig. 15(b). Figure 15(c) shows the sfPESI probe used for sampling and ionization. A microcentrifuge tube (T330-7LST, Simport, Qc, Canada) cut in half was used for the solvent reservoir. A stainless steel acupuncture needle was inserted into the gel loading tip with a protrusion of 0.1 mm from the tip. Water/methanol (1/1) solvent was used for the extraction of samples. About 1 mL solvent was prefilled in the reservoir with the liquid head of ∼30 mm. The outer surface of the gel loading tip was coated with a perfluoroalkyl film to make the surface hydrophobic.^15)^ This operation is mandatory to avoid carryover in the repetitive operation using a non-disposable single sfPESI probe (Fig. 15(c)).

In the sampling operation, the probe moved down at a speed of 0.1 mm s^−1^ to touch the sample surface. This slow speed of the sfPESI probe minimized the overrun of the probe system by the moment of inertia. When the probe touched the sample surface, the probe stopped there. This position was the starting point for the control of the invasion depth of the probe into the sample surface. Under normal operating conditions, the invasion depth of the probe was set to 0.0 mm to suppress the contamination of the probe tip with the sample. The contact time of the probe tip with the surface was also controlled from ∼50 ms to several tens of seconds. After sampling, the probe was transported in front of the inlet of the mass spectrometer by the 3-axis robot and a high voltage was applied to the needle of the probe for the acquisition of mass spectra.

**Figure 16:**
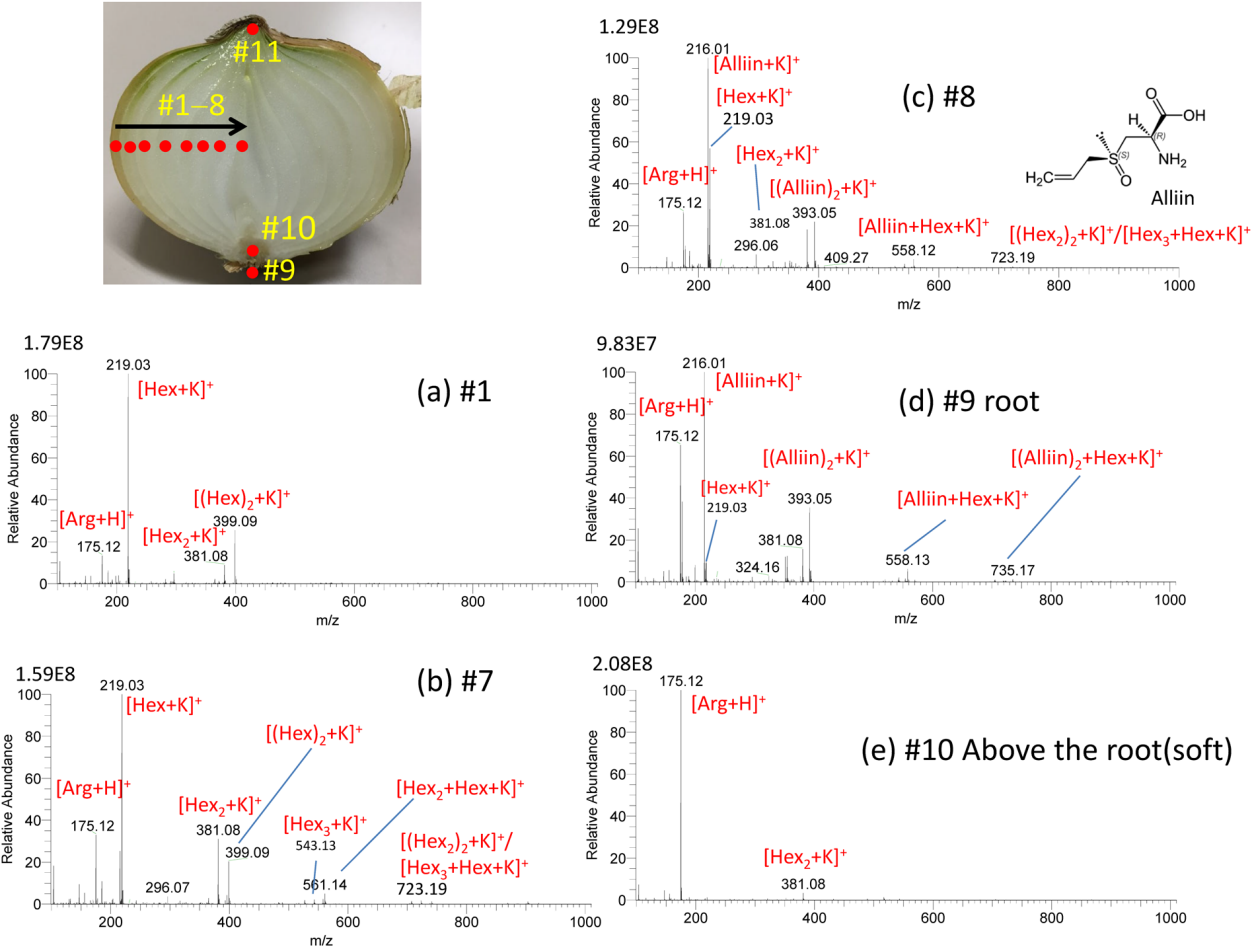
Fig. 16. sfPESI mass spectra of a sliced onion at positions of #1, and #7–#10 shown in the inset. Mass spectrum at #11 is similar to those at #8 and #9. Reprinted with permission from Ref. 46. Copyright (2020) American Chemical Society.

Robotic sfPESI has been applied to a variety of sample type. The leaf bases of the onion bulb can be regarded as a growth ring and are composed of old outer bases and young inner ones. The components and concentration of fructans vary from outer to inner bases. Figure 16 shows the results for the point analysis of a sliced onion.^46)^ A 3 mm-thick transverse section was cut midway between the top and bottom of the bulb and placed on the sample stage. The positions of the probe were changed stepwise from #1 to #11 by using the programming tool. No carryover was observed in the consecutive analysis of the positions #1→#11. In Fig. 16(a) at #1, [Arg+H]^+^, [Hex+K]^+^, and [(Hex)_2_+K]^+^ were detected as major ions with [Hex_2_+K]^+^ as a minor one. The relative intensities of Hex*_n_* (*n*≥2) to Hex increased with the change of the positions, #1→#7. As shown in Fig. 16(b) at #7, Hex*_n_* up to *n*=3 were detected. The concentration change is due to hydrolysis of fructans to free fructose which achieves osmotic adjustment as the base cells expand during bulbing of the onion. In addition, Hex functions in protecting the plant against water deficit as an osmoregulator. It should be noted that the mass spectrum showed a drastic change with #7→#8. At #8 as shown Fig. 16(c), potassiated alliin, its dimer, and mixed clusters of alliin and Hex*_n_* (*n*=1–3) in addition to [Arg+H]^+^ were detected as major ions. Alliin was only localized at the central part of the bulb. Figure 16(d) shows the mass spectrum for the root part at #9 that was similar to that at #8. However, the spongy soft part at #10 just above #9 (Fig. 16(e)) gave [Arg+H]^+^ as the dominant peak and [alliin+K]^+^, the base peak in #8 and #9, was barely detected. As described, this method is useful for the analysis of different sections of plants (profile analysis).

**Figure 17:**
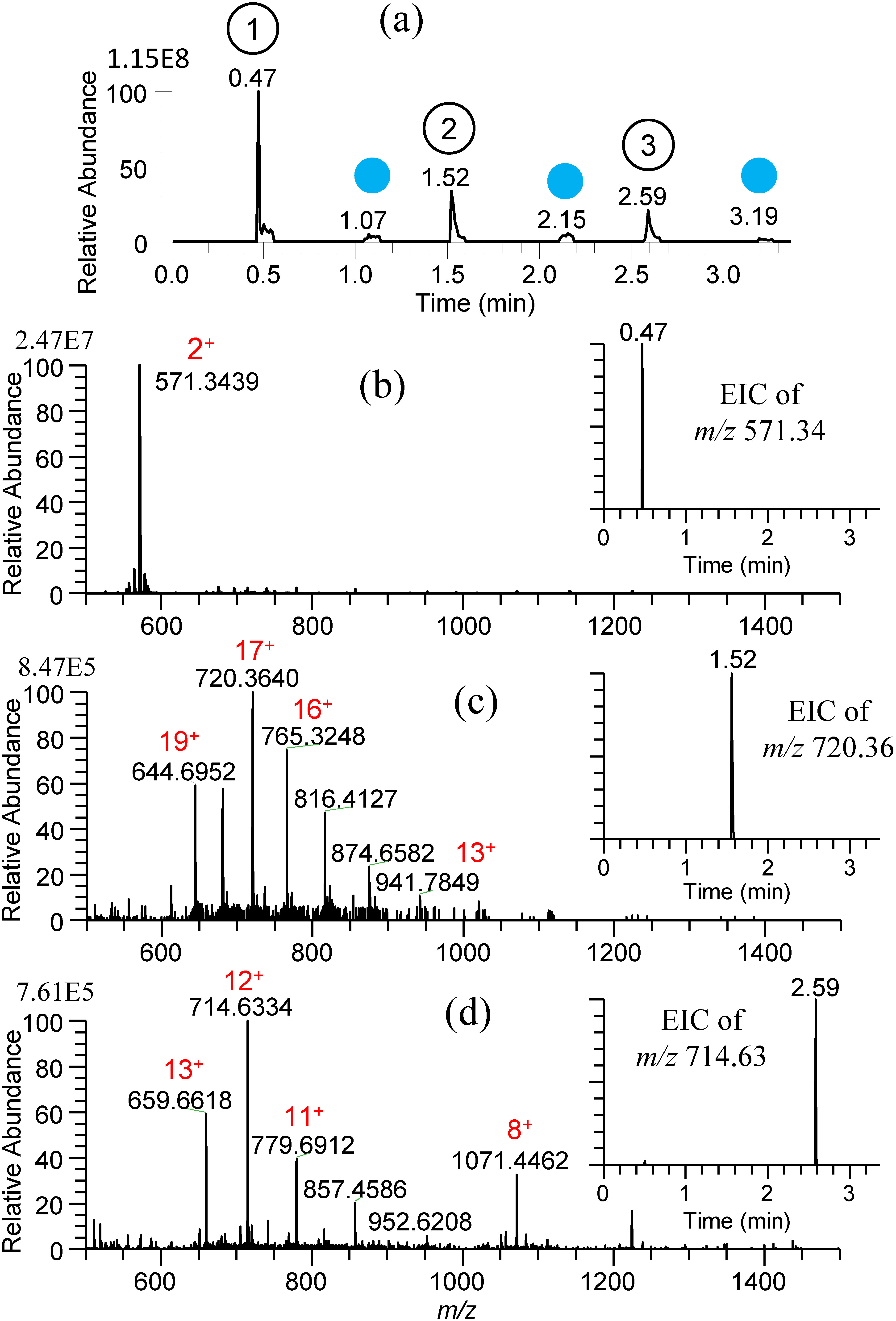
Fig. 17. (a) TIC for water/methanol (1/1) solutions of 1% acetic acid and 10^−5^ M gramicidin *S* (①), cytochrome *c* (②), and ubiquitin (③) prepared in the 96-well plate measured by robotic sfPESI equipped with a touch sensor. The probe was cleansed at 1.07, 2.15, and 3.19 min using water/methanol (1/1) solvent as shown in solid circles. (b) Mass spectrum for gramicidin *S* measured at 0.47 min. (b) Mass spectrum for cytochrome *c* measured at 1.52 min. (c) Mass spectrum for ubiquitin measured at 2.59 min. In EICs shown in the insets, little carryover was observed for these three samples. The contact time of the probe with the sample and the solvent for cleansing: ∼50 ms, invasion depth of the probe to the sample solution and to the solvent for cleansing: 0 mm. HV applied to the needle: 2.5 kV, HV duration time for the acquisition of the mass spectra: 5 s. Reproduced by permission of The Royal Society of Chemistry.

Figures 17(a)–(d) show the TIC and mass spectra for water/methanol (1/1) solutions of 10^−5^ M gramicidin *S*, cytochrome *c*, and ubiquitin prepared in the 96-well plate.^47)^ After each sampling, the sfPESI was cleansed by water/methanol solvent to remove the carryover. As shown in the insets of EICs for three samples, the carryover was negligible. Similar results were obtained for consecutive analysis of water/methanol solutions of 10^−5^ M cocaine, morphine, amphetamine, and methamphetamine giving protonated analytes [M+H]^+^ as major ions.

**Figure 18:**
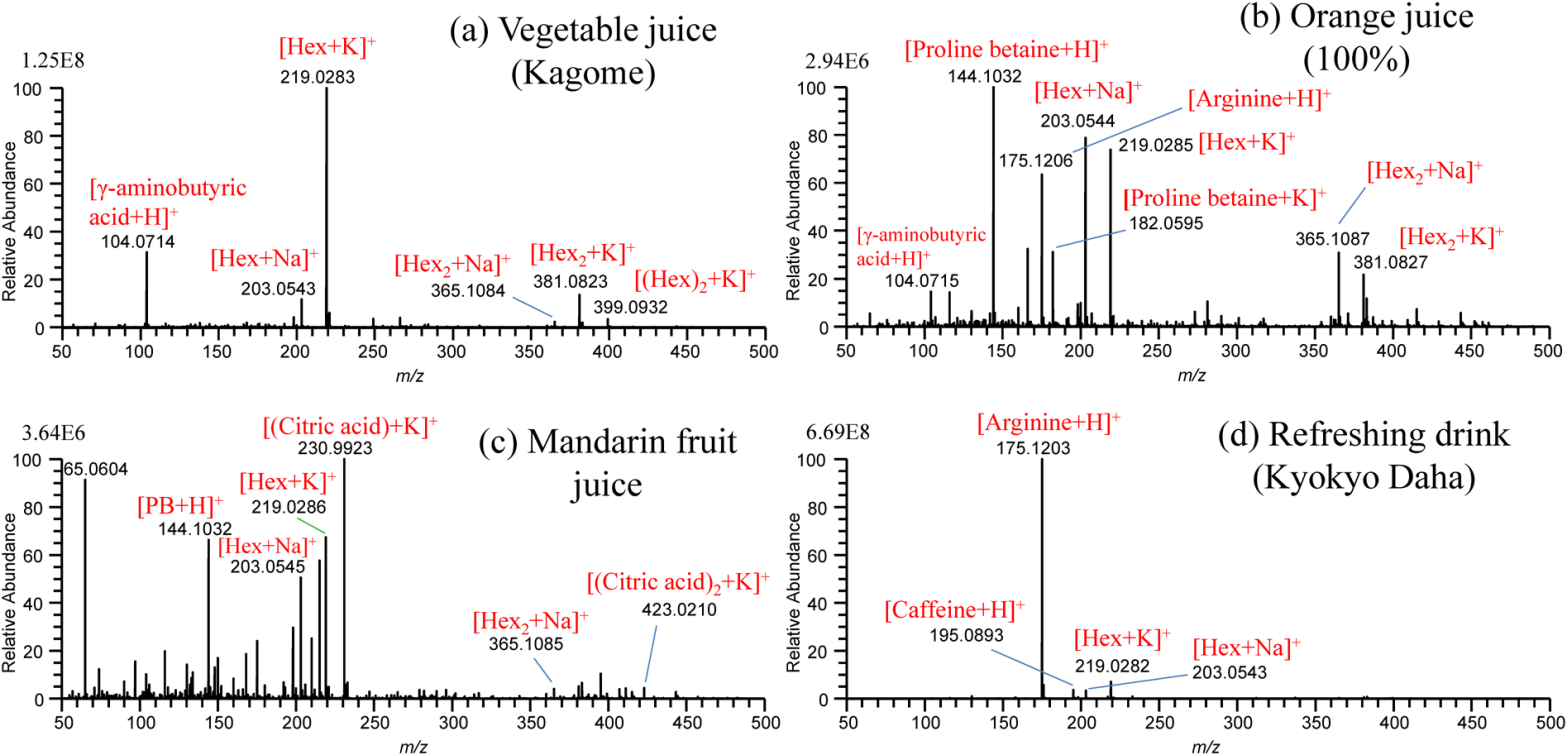
Fig. 18. Robotic/sfPESI mass spectra for undiluted real samples of (a) vegetable juice (Kagome Co., Ltd.), (b) orange juice (Seven Premium, Seven-Eleven Japan, Co., Ltd.), (c) squeezed juice of mandarin fruit (Citrus Unshiu)), and (d) refreshing drink (Kyokyo-Daha, Tokiwa Pharmaceutical, Co., Ltd.) filled in the multiwell plate. Reproduced by permission of The Royal Society of Chemistry.

For the practical application of the present method to undiluted real samples, vegetable juice (Kagome Co., Ltd.), orange juice (Seven Premium, Seven-Eleven Japan, Co., Ltd.), squeezed juice of mandarin fruit (Citrus Unshiu), and refreshing drink (Kyokyo-Daha, Tokiwa Pharmaceutical, Co., Ltd.), filled in a multiwell plate were examined.^47)^ Figures 18(a)–(d) show the mass spectra for these samples obtained by using water/methanol/acetonitrile (1/1/1) as the cleansing solvent. The peak at *m*/*z* 104.0714 in Fig. 18(a) for vegetable juice and Fig. 18(b) for orange juice was identified as γ-aminobutyric acid (GABA). Proline betaine as a biomarker of citrus was detected in orange juice and squeezed mandarin fruit. While [(citric acid)+K]^+^ was detected as the base peak for squeezed mandarin fruit juice in Fig. 18(c), it was detected at a trace level for orange juice in Fig. 18(b). The commercial orange juice may be manufactured from fully matured oranges. In Fig. 18(d), major components of arginine and caffeine contained in the refreshing drink were detected as major ions. The alleviation of carryover in sfPESI is due to the self-cleaning of the analytes captured at the probe tip by the flow of pure solvent prefilled in the sfPESI probe accompanied with the spontaneous electrospray.

## SOLVENT AND SOLUTION CHARACTERISTICS

PESI and related techniques can be applicable to a wide variety of samples as described above. Because PESI techniques rely on sample extraction by solvents, the choice of an appropriate solvent is a crucial consideration. Solutions and solvents used for PESI, dPESI, sfPESI, and ad-sfPESI are summarized in Table 2. In the table, water or aqueous organic solvents (methanol, ethanol and acetonitrile) for positive-mode, and aqueous 2-propanol for negative-mode PESI were used for the solvents. It is known that organic content above ∼80% can result in a decreased ESI response.^59,60)^ In other words, the use of “aqueous” organic solution is the key point for the standard electrospray operation. The reason for this is considered in the following. In ESI, the oxidation reactions in the positive mode, and reduction reactions in the negative-mode electrospray take place at the interface between the metal electrode and the solution. The excess charges generated at the interface are transported to the surface of the Taylor cone (*i.e.*, electrophoresis), electrosprayed toward the counter electrode, and are neutralized at the counter electrode after the flight in the ambient gas. That is, electrospray takes advantage of “half-cell” for the generation of charged droplets liberated in the gas phase. The oxidation or reduction reactions anticipated to take place at the metal electrodes are,^25)^

oxidation (positive-ion mode):
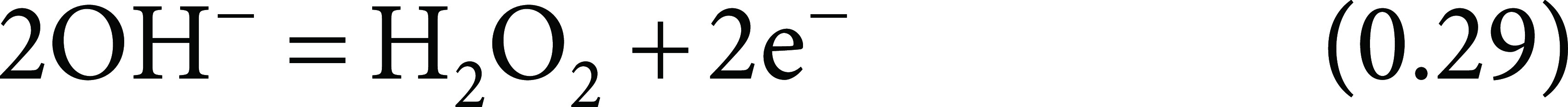
(8)
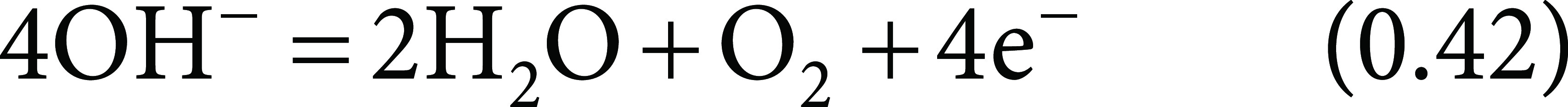
(9)
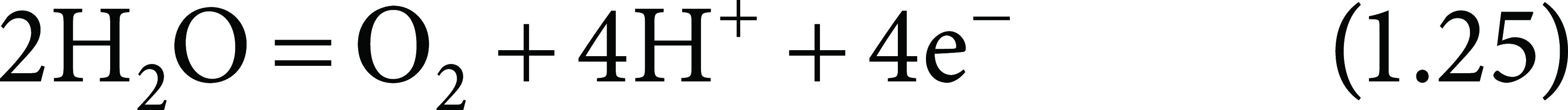
(10) reduction (negative-ion mode): 
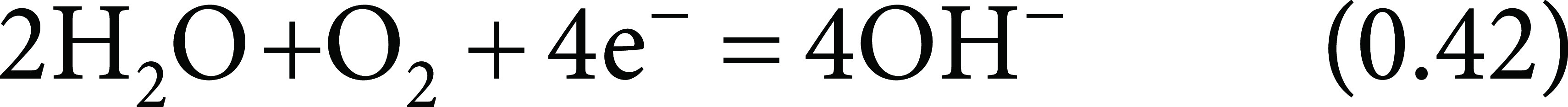
(11) where the number in bracket stands for *E*_0_ (V *vs.* 2H^+^/H_2_). The decrease in [OH^−^] by reactions (8) and (9) concomitantly leads to the increase in [H^+^] by the equilibrium of [H^+^][OH^−^]=10^−14^. Reactions (8)–(11) manifest that water in aqueous solution plays the major role for the generation of excess charges, H^+^ in the positive-mode and OH^−^ in the negative-mode ESI. The excess charges of H^+^ or OH^−^ generated at the interface will drift to the surface of the Taylor cone under the influence of a strong electric field exerted at the tip of the capillary electrode, *i.e.*, electrophoresis of the excess charges in the solution. The electrical conduction in solution is governed by the movement of ions in liquid medium. Here, it should be pointed out that H^+^ and OH^−^ have exceptionally high mobilities in water compared to other ions, *e.g.*, the mobilities of ions in water (×10^−8^ m^2^ s^−1^ V^−1^) are H^+^(36.2), NH_4_^+^(7.6), Na^+^(5.2), K^+^(7.6), OH^−^(20.5), and Cl^−^(7.9). Due to the high mobilities of H^+^ and OH^−^ in water, aqueous solution has the lower internal resistance for the charge carriers of H^+^ or OH^−^ resulting in more rapid electrochemical reactions on the metal electrode interface than other pure organic solvents. The abnormally high mobilities of H^+^ and OH^−^ have been interpreted in terms of sequence of proton-transfer reactions; proton hopping between water molecules (Grotthuss mechanism).^61)^ In short, water in aqueous organic solution acts as the generation of excess charges (H^+^ or OH^−^) and also plays a role for the best medium for the transportation of excess charges in solution (electrophoresis). Besides, aqueous organic solvents are beneficial for PESI because of slow evaporation of sample solution compared to more volatile pure organic solvents.

**Table 2:** Table 2. Solutions and solvents for sample extraction for PESI, dPESI, sfPESI and ad-sfPESI.

Method	Sample	Solvent for extraction	Reference
PESI			
(+)	10^−5^ M gramicidin *S*+melittin in H_2_O		11
	10^−4^ M cytochrome *c*+gramicidin *S* +10^−2^M AcONH_4_ in H_2_O		
	10^−4^ M cytochrome *c* +10^−2^M AcOH in H_2_O		
	10^−5^ M insulin +0.1%TFA in H_2_O		
	0.2 g/L PEG 2000+10^−3^ M NaCl inH_2_O		
	Soft drink		
(+)	10^−5^ M gramicidin *S*+10^−2^M AcONH_4_ in H_2_O		24
	10^−6^M gramicidin *S*+10^−2^M AcONH_4_ in H_2_O		
	10^−6^M gramicidin *S*+0.1% AcOH in H_2_O		
	10^−5^ bovine insulin +0.1% AcOH in H_2_O		
(+)	human milk, cow’s milk, beer, banana, onion, mouse brain, flower petal		48
(+)	mouse urine, serum, homogenized liver		23
(+)	tulip bulb, tulip petal		41
(+)	4.8×10^−5^ M myoglobin+glacial AcOH in H_2_O/MeOH(75/25)		32
	10^−5^ M gramicidin *S* in H_2_O/MeOH/AcOH(1/1/0.0025)+D_2_O/CH_3_OD		
	10^−2^ M benzaldehyde in H_2_O/MeOH(3/7)+10^−1^ M ethanolamine in H_2_O/MeOH(3/7)		
(+)	10^−5^M gramicidin *S*+1% AcOH +1 M NaCl in MeOH/H_2_O(25/75)		12
	10^−5^M myoglobin +0–4 M urea in MeOH/H_2_O(25/75)		
	10^−5^M myoglobin +5–500 mM NaCl in MeOH/H_2_O(25/75)		
	10^−5^M myoglobin +5–500 mM K_3_PO_4_ in MeOH/H_2_O(25/75)		
	10^−5^M gramicidin *S*+0–1 M K_3_PO_4_ in MeOH/H_2_O(25/75)		
(+)	10^−3^ M Triton X100+10^−5^ M cytochrome *c* in H_2_O/MeOH/AcOH(74/25/1)		13
	1.5×10^−2^ M NaCl +10^−5^ M insulin in H_2_O/MeOH/AcOH (74/25/1)		
	10^−5^ M cytochrome *c* +10^−5^ M L-α-phosphatidylcholin (egg yolk) in H_2_O/MeOH/AcOH (74/25/1)		
	human breast cancer	10 μL H_2_O/MeOH/AcOH (50/50/1) dropped on the tissue	
(+)	10^−5^ M gramicidin *S*+1 M NaCl in H_2_O/MeOH/AcOH(75/25/1)		49
	10^−5^ M gramicidin *S*+1 M phosphate buffer in H_2_O/MeOH/AcOH(75/25/1)		
	10^−5^ M myoglobin +250 mM NaCl in H_2_O/MeOH/AcOH(75/25/1)		
	10^−5^ M myoglobin +4 M urea in H_2_O/MeOH/AcOH(75/25/1)		
	10^−5^ M myoglobin +250 mM phosphate buffer in H_2_O/MeOH/AcOH(75/25/1)		
(+)	clear cell renal cellcarcinoma (homogenized in 0.9% aqueous NaCl)		34
(+, −)	10^−4^ rhenium organo metalics in MeOH		50
(+)	illicit drugs in urine, oral fluid, plasma		51
(+)	0.4 M copper(II)+3.0 M lactate +3.7 M NaOH in H_2_O		28
(−)	10^−5^ M insulin, cytocrome *c*, ubiquitin in H_2_O/2-PrOH(1/1)		52
	10^−6^ M angiotensin II, 5´10^−6^ M angiotensin I, gramicidin *S*, Asp-Asp-Asp-Asp,		
	5×10^−6^ M angiotensin I, gramicidin *S*, Lys-Lys-Lys-Lys-Lys, substance P,		
	in H_2_O/2-PrOH(1/1)		
	10 ppm L-α-phosphatidylinositol, 1,2-diacyl-*sn*-glycero-3-phospho-L-serine		
	in H_2_O/2-PrOH(1/1)		
	cancerous human colon	10 μL H_2_O/2-PrOH dropped on the tissue	
(+)	5×10^−5^ M cholesterol +10^−5^ M cytochrome *c* in H_2_O/MeOH/FA(1/1/0.001)		53
(+)	sobean, walnut	10 μL H_2_O/MeOH dropped on the surface	54
(+)	mouse brain (imaging)	160°C H_2_O or H_2_O/MeCN(7/3) vapor	30
(+)	liver of control/steatotic mice	160°C chloroform/MeOH (7/3) vapor	33
(+)	pepsin/tripsin +10^−5^ M cytochrome *c* in H_2_O (pH=1–8)	120°C H_2_OMeCN(1/1) vapor	31
(+)	normal/cancer kidney	150°C H_2_O/MeCN(1/1) vapor	55
(+)	single cell (rhodendron petal, soybean sprout)	H_2_O/MeCN(1/1) micro droplets, piezo inkjet generator	39
dPESI			
(+)	tomato, spinach, onion, salmon flesh, cow’s milk, yogurt, soybean milk	H_2_O/MeOH(1/1)	14
sfPESI			
(+)	tablets, bill, fruits, potato, narcotics, 100 pg–100 ng for codeine (linear relation)	MeOH	56
(+)	acephate, chlothianidin, acetamiprid, thiophanate-methyl on living plants	H_2_O/MeCN/FA(1/1/0.001)	57
(+)	ball point pen, coffee powder, tablets, rice grain, narcotics, dried rhodamine B	H_2_O/MeOH/MeCN(1/1/1)	15
(+)	fresh blood, saliva and urine, dried blood	H_2_O/EtOH(1/1)	58
(+)	strawberry, browning apple, onion (scales, root, leaf)	H_2_O/MeOH(1/1)	46
(+) (robotic)	commercial vegetable, orange and refreshing drink prepared in a multiwell plate	H_2_O/MeOH(1/1)	47
ad-sfPESI			
(+)	apple, strawberry, tomato (leaf, stem, premature, ripened), camellia, sweet potato, apricot (premature, ripened, seed), orange (oil gland, juice), potato (sprout, flesh, leaf)	H_2_O/MeOH(1/1)	16

AcOH: acetic acid, TFA: trifluoroacetic acid, AcONH_4_: ammonium acetate, MeOH: methanol, EtOH: ethanol, 2-PrOH: 2-propanol, MeCN: acetonitrile, FA: formic acid, (+): positive mode, (−): negative mode.

As shown in Table 2, methanol, ethanol, and acetonitrile were used as the appropriate solvents for the aqueous organic solvents in the positive-mode PESI. In contrast, aqueous 2-propanol was used in the negative-mode PESI.^52)^ The use of 2-propanol is not recommended for the positive-mode electrospray because the cluster ions of [(2-propanol)*_n_*+H]^+^ prevail in the mass spectra and they severely suppress the analyte ion signals. In contrast in the negative-mode operation, aqueous 2-propanol gave satisfactory mass spectra of analytes.^52)^ It was found that the threshold voltage of electrospray for aqueous 2-propanol is lower than those for aqueous methanol and ethanol resulting in the suppression of corona discharge. According to Eq. (4), the better performance of aqueous 2-propanol than aqueous methanol and ethanol is likely to be due to the lower surface tension γ of the former than the latters. The values of surface tensions (10^−3^ N m^−1^) for water, methanol, ethanol, and 2-propanol at 25°C are 71.85, 22.07, 21.97, and 20.93, respectively. That is, γ values for methanol, ethanol, and 2-propanol are of the same order. However, this may not be the case for aqueous alcohols. Because isopropyl groups are more hydrophobic than methyl or ethyl groups, 2-propanol may be more enriched on the surface of the aqueous 2-propanol than methanol and ethanol, resulting in the decrease of surface tension, *i.e.*, threshold voltage of electrospray.

## QUANTITATIVE EVALUATIONS

As described above, PESI techniques are ideally suited to qualitative biochemical analysis because they allow applications to any states of the samples (liquid, viscous, or solid). Because of the operational simplicity, significantly more rapid, direct and sensitive analyses can be achieved compared to conventional capillary-based electrospray. Finally, quantitative evaluation of PESI techniques will be described in brief. The performance of electrospray is affected by so many factors such as surface tension of the sample, applied voltage, solvent, additives in solution, surface activity of each analyte ion, and even change of atmospheric pressure and relative humidity of the ambient air. Thus, the reliable quantitative analysis may only be performed with the use of an internal standard (IS). Saha *et al.* evaluated the quantitative capabilities of PESI from urine, oral fluid and plasma using methamphetamine as a representative illicit drug and *N*-benzylmethylamine as an internal standard.^51)^ The limits of detection for illicit drugs in body fluids were one to two orders of magnitude higher compared to the standard samples and the relative standard deviations for the quantitative analysis were found to be less than 20%. Rahman *et al.* examined the quantitative aspect of sfPESI using a standard sample^56)^ (no IS used). In order to assess the response linearity, known amounts of codeine dissolved in methanol were deposited/dried on the non-sticky side of the scotch tape, and the central part of the deposited area was examined. It was found that a nearly straight line for plots between the amounts of codeine in the range of 100 pg to 100 ng and signal intensities was obtained. Linear response was also observed for standard samples of pesticides by sfPESI.^57)^

## CONCLUSION

As described, PESI and its related techniques are useful in various respects, *e.g.*, ambient ionization, minimal or no sample preparation, small sample amounts, high detection sensitivity, non-invasive sampling, *in situ* sampling, on site remote sampling, imaging, single cell observation, resistivity to high salt/detergent concentration, minimal suppression effect, and automation by the robotic system. These unique characteristics originate from the fact that PESI and its relatives rely on discontinuous sampling followed by spontaneous electrospray. That is, sequential and exhaustive electrospray takes place in the order of surface-active values of analytes. PESI techniques would be promising for mass spectrometry in the next generation.

## Notes

Mass Spectrom (Tokyo) 2020; 9(1): A0092
